# Combined Field Inoculations of *Pseudomonas* Bacteria, Arbuscular Mycorrhizal Fungi, and Entomopathogenic Nematodes and their Effects on Wheat Performance

**DOI:** 10.3389/fpls.2017.01809

**Published:** 2017-10-31

**Authors:** Nicola Imperiali, Xavier Chiriboga, Klaus Schlaeppi, Marie Fesselet, Daniela Villacrés, Geoffrey Jaffuel, S. Franz Bender, Francesca Dennert, Ruben Blanco-Pérez, Marcel G. A. van der Heijden, Monika Maurhofer, Fabio Mascher, Ted C. J. Turlings, Christoph J. Keel, Raquel Campos-Herrera

**Affiliations:** ^1^Department of Fundamental Microbiology, University of Lausanne, Lausanne, Switzerland; ^2^FARCE Laboratory, University of Neuchâtel, Neuchâtel, Switzerland; ^3^Plant-Soil-Interactions, Department of Agroecology and Environment, Agroscope Reckenholz, Zurich, Switzerland; ^4^Plant Breeding and Genetic Resources, Institute for Plant Production Sciences, Agroscope Changins, Nyon, Switzerland; ^5^Department of Land, Air, and Water Resources, University of California, Davis, Davis, CA, United States; ^6^Institute of Integrative Biology, ETH Zurich, Zurich, Switzerland; ^7^Centro para os Recursos Biológicos e Alimentos Mediterrânicos (MeditBio), Universidade do Algarve, Faro, Portugal; ^8^Department of Evolutionary Biology and Environmental Studies, University of Zurich, Zurich, Switzerland; ^9^Plant-Microbe Interactions, Faculty of Science, Institute of Environmental Biology, Utrecht University, Utrecht, Netherlands

**Keywords:** plant-growth promoting rhizobacteria, biofertilizer, *Steinernema*, *Heterorhabditis*, wheat, biological control, insect pest, plant growth promotion

## Abstract

In agricultural ecosystems, pest insects, pathogens, and reduced soil fertility pose major challenges to crop productivity and are responsible for significant yield losses worldwide. Management of belowground pests and diseases remains particularly challenging due to the complex nature of the soil and the limited reach of conventional agrochemicals. Boosting the presence of beneficial rhizosphere organisms is a potentially sustainable alternative and may help to optimize crop health and productivity. Field application of single beneficial soil organisms has shown satisfactory results under optimal conditions. This might be further enhanced by combining multiple beneficial soil organisms, but this remains poorly investigated. Here, we inoculated wheat plots with combinations of three beneficial soil organisms that have different rhizosphere functions and studied their effects on crop performance. Plant beneficial *Pseudomonas* bacteria, arbuscular mycorrhizal fungi (AMF), and entomopathogenic nematodes (EPN), were inoculated individually or in combinations at seeding, and their effects on plant performance were evaluated throughout the season. We used traditional and molecular identification tools to monitor their persistence over the cropping season in augmented and control treatments, and to estimate the possible displacement of native populations. In three separate trials, beneficial soil organisms were successfully introduced into the native populations and readily survived the field conditions. Various *Pseudomonas*, mycorrhiza, and nematode treatments improved plant health and productivity, while their combinations provided no significant additive or synergistic benefits compared to when applied alone. EPN application temporarily displaced some of the native EPN, but had no significant long-term effect on the associated food web. The strongest positive effect on wheat survival was observed for *Pseudomonas* and AMF during a season with heavy natural infestation by the frit fly, *Oscinella frit*, a major pest of cereals. Hence, beneficial impacts differed between the beneficial soil organisms and were most evident for plants under biotic stress. Overall, our findings indicate that in wheat production under the test conditions the three beneficial soil organisms can establish nicely and are compatible, but their combined application provides no additional benefits. Further studies are required, also in other cropping systems, to fine-tune the functional interactions among beneficial soil organisms, crops, and the environment.

## Introduction

In addition to poor soil fertility, soil pests and pathogens pose major threats to the health and productivity of crops in agricultural ecosystems resulting in important yield losses every year (Oerke, 2006; Kupferschmied et al., 2013). The use of fertilizers, fungicides, nematicides, and insecticides to counter these problems can have important negative consequences, such as the persistence of these agrochemicals in the soil, water, and food with potential negative impacts on the environment and consumers (Bale et al., 2008; Lichtfouse et al., 2009; Kupferschmied et al., 2013; Johnson et al., 2016). Hence, new and more sustainable pest and disease control strategies need to be explored for a next-generation agriculture and the application of beneficial soil organisms (BeSO) presents a promising alternative for maintaining crop health and productivity (Bommarco et al., 2013; Bender et al., 2016).

Various BeSO are known to enhance plant performance, e.g., by directly promoting plant growth, by stimulating plant defenses, by facilitating nutrient acquisition by the plant, or by protecting the plant from pathogens and pests (Philippot et al., 2013; Rasmann and Turlings, 2016; Venturi and Keel, 2016). The three groups of BeSO investigated in the present study fulfill one or several of these beneficial functions, i.e., plant-growth promoting rhizobacteria (PGPR), arbuscular mycorrhizal fungi (AMF), and entomopathogenic nematodes (EPN). Root-colonizing bacteria belonging to the *Pseudomonas fluorescens* group are well-characterized PGPR that have the ability to induce systemic plant defenses and ward off soil-borne pathogens, in particular pathogenic fungi and oomycetes, including *Gaeumannomyces, Thielaviopsis, Rhizoctonia, Fusarium oxysporum*, and *Pythium* (Haas and Défago, 2005; Mercado-Blanco and Bakker, 2007; Hol et al., 2013; Vacheron et al., 2015). To date, several biocontrol products that are based on PGPR pseudomonads are on the market (Berg, 2009; Kupferschmied et al., 2013). Moreover, certain subgroups, in particular the two species *Pseudomonas protegens* and *Pseudomonas chlororaphis* exhibit potent oral insecticidal activity notably against Lepidopteran pests (Kupferschmied et al., 2013; Ruffner et al., 2013; Flury et al., 2016).

AMF are well-known beneficial symbionts that colonize the roots of the majority of land plants (Schueßler et al., 2001; van der Heijden et al., 2015). AMF form extensive hyphal networks that provide water and nutrients to their host plant. AMF are key actors in processes such as the mineralization of phosphorus and nitrogen and enhancing the nutrient up-take by plant roots (Jakobsen et al., 1992; Mäder et al., 2000; Smith et al., 2004; van der Heijden et al., 2006). AMF primarily improve plant nutrition, but they can also contribute to enhance the tolerance of their host plant against biotic and abiotic stresses (van der Heijden et al., 2015). Numerous AMF species, e.g., *Rhizoglomus irregulare*, are commercialized as inoculum to improve soil fertility (Lekberg and Koide, 2005; Pellegrino et al., 2015) and plant productivity (Hijri, 2016; Köhl et al., 2016). Today, the agronomic use of AMF includes the direct augmentation or inoculation of seedlings in nurseries before transplanting to the field (Jeffries et al., 2003) and seed coating (e.g., Ijdo et al., 2011).

Finally, EPN of the genera *Steinernema* and *Heterorabditis* are well-known biocontrol agents that selectively search their insect hosts and kill them within 2–3 days with the aid of mutualistic bacteria of the genera *Xenorhabdus* and *Photorhabdus*, respectively (Georgis et al., 2006; Kaya et al., 2006; Dillman et al., 2012; Campos-Herrera, 2015; Lacey et al., 2015). Their wide distribution in soils throughout the world (Adams et al., 2006) and the availability of commercial products (Lacey et al., 2015) make them excellent products in integrated pest management (IPM) programs or in organic production, both for augmentation or restoration of naturally occurring EPN (Campos-Herrera, 2015). However, their performance and activity is affected by biotic and abiotic factors, and hence, their efficacy depends on soil characteristics, agricultural management practices, and competition within the food web (Stuart et al., 2015).

The three groups of organisms—*Pseudomonas*, AMF, and EPN—occur naturally in most arable soils and commercial formulations are available for agronomic use (Stockwell and Stack, 2007; Berg, 2009; Kupferschmied et al., 2013; Lacey et al., 2015). Previous greenhouse and field studies have reported varying effects on plant health and growth when combining inoculants of these BeSO groups. For example, combinations of certain *Pseudomonas* strains provided better control of the wheat disease take-all than did the individual strains alone (Pierson and Weller, 1994). Positive effects have been also recorded when combining bacteria, such as *Pseudomonas* or *Azospirillum* strains, with fungi, including the AMF *Glomus* (Frey-Klett et al., 2007; Couillerot et al., 2012; Walker et al., 2012), *Fusarium* or *Trichoderma* (Fogliano et al., 2002; Yigit and Dikilitas, 2007). Similarly, EPN have been combined with other BeSO, with differing results. For example, the combination of *Steinernema kraussei* with the entomopathogenic fungus (EPF) *Metarhizium anisopliae* resulted in a synergistic effect in the control of *Otiorhynchus sulcatus* in strawberry (Ansari et al., 2010), while the combination of *Steinernema ichnusae* with the EPF *Beauveria bassiana* resulted in clear antagonism and competition for the host under controlled laboratory conditions (Tarasco et al., 2011).

Field applications of single BeSO have shown to greatly enhance plant growth and health in various crops (Jeffries et al., 2003; Berg, 2009; Kupferschmied et al., 2013; Campos-Herrera, 2015; Lacey et al., 2015), but the putative positive effect of combining various BeSO remains poorly predictable. The Swiss National Research Programme 68 (NRP 68) “Sustainable use of soil as a resource” (www.nrp68.ch) provided the framework for our multidisciplinary investigations into BeSO and their possible role in novel strategies for sustainable soil management. As part of this, we evaluated, for the first time, the simultaneous application of *Pseudomonas*, AMF, and EPN inoculants in field experiments, using wheat as the model crop. We hypothesized that the combined application of these BeSO would show greater benefits for the crop than their individual application.

## Materials and methods

### Beneficial organisms

Selected species of BeSO, all known to naturally occur in Swiss soils (Campos-Herrera et al., 2015a; Jaffuel et al., 2016; Schlaeppi et al., 2016; Imperiali et al., 2017), were applied depending on the objective and design of each field experiment (Figure S1). The BeSO that were used included two species of the genus *Pseudomonas*, three AMF species and four EPN species and they were applied as inoculants either individually or in various combinations in the different experiments (Tables 1, 2).

**Table 1:** Beneficial soil organisms applied individually or in combinations in the field experiments.

**Beneficial group/species**	**Strain**	**Treatment code**	**Application type**	**GenBank accession no**.	**Reference or source**
***PSEUDOMONAS*** **BACTERIA**					
*Pseudomonas chlororaphis*	PCL1391^a^	B2	Aqueous	NZ_LFUT01000004	Chin-A-Woeng et al., 1998
*Pseudomonas protegens*	CHA0^a^	B1	Aqueous	NC_021237	Stutz et al., 1986
**ARBUSCULAR MYCORRHIZAL FUNGI**					
*Claroideoglomus claroideum*	SAF12^c^	F4	Substrate	n.a.^d^	Swiss collection of arbuscular miychorrizal fungi (SAF)
*Funneliformis mosseae*	SAF11^c^	F3	Substrate	n.a.^d^	SAF
*Rhizoglomus irregulare*^b^	INOQ Top	F1	Substrate	n.a.^d^	Inog GmbH, Schnega
*Rhizoglomus irregulare*^b^	SAF22^c^	F2	Substrate	DQ377990	Germany, SAF
**ENTOMOPATHOGENIC NEMATODES**					
*Heterorhabditis bacteriophora*	Andermatt	N2	Aqueous	KJ938576	Andermatt Biocontrol AG, Grossdietwil, Switzerland
*Heterorhabditis megidis*	Andermatt	N1	Aqueous	KJ938577	Andermatt Biocontrol AG, Grossdietwil, Switzerland
*Steinernema carpocapsae*	D-83	N3	Aqueous	KJ818295	Jaffuel et al., 2016
*Steinernema feltiae*	RS-5	N4	Aqueous	KJ938569	Jaffuel et al., 2017

a *Rifampicin-resistant variants of strains CHA0 and PCL1391 were used as inoculants in the field trials (see Materials and Methods)*.

b *Rhizoglomus irregulare was previously referred to as Rhizophagus irregularis and earlier as Glomus intraradices (Sieverding et al., 2015)*.

c *Strain ID referring to the Agroscope AMF strain collection, http://www.agroscope.ch/saf*.

d *n.a., not available*.

**Table 2:** Details on the characteristics of the three field experiments used to assess effects of inoculation of beneficial soil organisms (pseudomonads, entomopathogenic nematodes, and arbuscular mycorrhizal fungi) on growth, health, and yield of spring wheat.

**Field trials**	**COMBINATION**	**PERFORMANCE-1**	**PERFORMANCE-2**
Coordinates SN/EW	46.397676/6.260763	46.397455/6.260166	46.39502/6.260444
Sowing/inoculation day	18th March 2014	18th March 2014	27th March 2015
Treatments (Treatments codes refer to Table 1)	Control (no inoculants) B1: *P. protegens* CHA0-Rif B2: *P. chlororaphis* PCL1391-Rif N1: *H*. *megidis* Andermatt N2: *H. bacteriophora* Andermatt N3: *S. carpocapsae* D83 N4: *S. feltiae* RS5 F1-H: *R. irregulare* INOQ Top (high dosage, 250 ml/row) F1-L: *R. irregulare* INOQ Top (low dosage, 50 ml/row) F2: *R. irregulare* SAF22 F3: *F. mosseae* SAF11 F4: *C. claroideum* SAF12 AMF control (substrate only) B1+ N2 B1+ F1 N2 + F1 B1+ N2 + F1	Control (no inoculants) B1: *P. protegens* CHA0-Rif B2: *P. chlororaphis* PCL1391-Rif N2: *H. bacteriophora* Andermatt BM: B1 + B2 B1 + N2 B2 + N2 BM + N2	Control (no inoculants) BM: B1 + B2 NM: N1 + N2 + N4 F1-L AMF control (substrate only) BM + NM NM + F1-L BM + F1-L BM + NM + F1-L
Number of treatments	17	8	9
Number of replicates (plots) per treatment	Four replicates in randomized complete block design	Four replicates in randomized complete block design	Nine replicates in randomized complete block design
Size of plots	1.5 m^2^	6.75 m^2^	9 m^2^
Number of plant rows per plot	5	5	5
Wheat seeds per m of row	~80	~140	~140
Bacterial inoculum (CFU in 400 ml H_2_O per meter of row)	B1: 1.19 × 10^9^ B2: 1.21 × 10^9^	B1: 1.42 × 10^9^ B2: 3.37 × 10^9^	B1: 4.875 × 10^8^ B2: 8.25 × 10^8^
AMF inoculum	F1-H: 80 F1-L: 16 F2: 250 F3: 250 F4: 250 AMF control: 250	Not contributed	F1-L: 16 AMF control: 16
Nematode inoculum	50 infective juveniles/cm^2^/4 L	50 infective juveniles/cm^2^/8 L	50 infectice juveniles/cm^2^/8 L
Pest insect stress	Heavy natural infestation with *Oscinella frit*	Heavy natural infestation with *Oscinella frit*	No relevant *Oscinella frit* infestation
Soil type and texture	Sandy loam (clay, 25.5%; silt, 34.3%; sand; 40.2%)	Sandy loam (clay, 25.5%; silt, 34.3%; sand, 40.2%)	Loam (clay, 14.5%; silt, 26.8%; sand, 58.7%)

To monitor the bacteria following field application, the bacterial inoculants, i.e., *P. protegens* strain CHA0 (Stutz et al., 1986) and *P. chlororaphis* strain PCL1391 (Chin-A-Woeng et al., 1998) were tagged with a spontaneous resistance to rifampicin following previously described protocols (Natsch et al., 1994). Briefly, spontaneous rifampicin-resistant derivatives were obtained following plating concentrated cell suspensions of each parental strain on King's medium B agar (KMB) (King et al., 1954) supplemented with 100 μg/ml of rifampicin and incubated for 3 days. A CHA0-Rif derivative and a PCL1391-Rif derivative (Table 1), which stably maintained rifampicin resistance and displayed wild-type growth and antifungal and insecticidal activities, were selected. For the preparation of the bacterial field inocula, the selected rifampicin resistant strains were grown overnight at 25°C in lysogeny broth (LB) (Bertani, 1951) containing 100 μg/ml of rifampicin. Aliquots of 200 μl of each culture were spread on KMB plates without antibiotics. After incubation at 27°C for 16 h, bacterial cells were harvested and washed in sterile distilled water. The optical density at 600 nm (OD_600_) of the bacterial cell suspensions was adjusted to 0.15 corresponding to a cell density of 8 × 10^7^ CFU ml^−1^. These bacterial stock suspensions were maintained on ice until final dilution and use on the field sites.

The AMF strains *Claroideoglomas claroideum* SAF12, *Funneliformis mosseae* SAF11 and *R. irregular*e SAF22 were selected from the Swiss Collection of Arbuscular Mycorrhizal Fungi (SAF) at Agroscope (Reckenholz, Zurich, www.agroscope.ch/saf; Table 1). The inocula were prepared as described by Schlaeppi et al. (2016). Briefly, AMF were propagated over 6 months in the greenhouse in autoclaved sand:soil (85:15%; v/v) as substrate and using *Plantago lanceolata* as host. The final inoculum contained pieces of plant roots mixed with the substrate containing AMF hyphae and spores (SAF12, Propagation 0510, *P. lanceolata* roots were colonized by 28% and 763 spores could be washed from 25 g substrate; SAF#11, Propagation 0711, 27% and 29 spores; SAF#22, Propagation 0813; 97% and 475 spores). In addition, a “mock” inoculum consisting of *Plantago* roots and substrate free of AMF propagules was prepared following the same protocol and this mock treatment was termed “AMF control” (Quality inspection of the mock inoculum, Propagation 0711, roots were not colonized by AMF and no spores could be washed from 25 g of substrate). The COMBINATION experiment also comprised a treatment where nothing was applied, named “control”. AMF inocula as well as the mock-inoculum were mixed in separate plastic bags and stored at room temperature until use. In addition, the commercial AMF inoculum *R. irregulare* TOP (INOQ GmbH, Schnega, Germany, www.inoq.de) was used as obtained from Otto Hauenstein Samen AG (Rafz, Switzerland, www.hauenstein.ch). For the second trial (PERFORMANCE-2), we utilized the lower dosage of the commercial inoculum based on the COMBINATION experiment results. For the PERFORMANCE-2 trial we utilized autoclaved commercial inoculum as AMF control treatment.

For the EPN, infective juveniles (IJs) of four species were prepared in adjusted suspensions. *Heterorhabiditis* species were obtained from a commercial source (Andermatt Biocontrol, Grossdietwil, Switzerland, www.andermattbiocontrol.com), whereas *Steinernema* species were propagated from field collected populations under laboratory conditions following protocols described by Campos-Herrera et al. (2015a) (Table 1). All nematodes were received or harvested within 2 weeks prior to field application. The day before application, the EPN inoculant suspensions were prepared in sterile water. To this end, IJs were counted and their density was adjusted to deliver the required field concentration per experimental unit (Table 2) by using separate containers. Containers were kept at 5°C overnight and transported in coolers to the field. In addition, laboratory infections of *Galleria mellonella* larvae by the inoculant EPN at field concentrations were used to verify their infectivity for each experiment (Jaffuel et al., 2017).

### Experimental designs

From spring 2014 to summer 2015, three field experiments were conducted in wheat plots and the applications of beneficial soil organisms were adapted for each experiment. All the experiments were carried out with the commercial spring wheat variety “Rubli” in the experimental plots, whereas the commercial triticale variety “Trado” was seeded in the buffer zones. Fields were bordered by strips of non-managed grassland. The three experiments were named as follows: COMBINATION (2014), PERFORMANCE-1 (2014), and PERFORMANCE-2 (2015) (Table 2). The selection of the applied organisms and combinations of treatments were adapted on results of the preceding trial. The first experiment (COMBINATION) was set up to test various species of each group of beneficials and first combinatory treatments. The second experiment was designed to evaluate wheat yield effects after combining bacteria and EPN (PERFORMANCE-1). In this experiment, the AMF treatment was not included due to limitations in scaling the production of inoculum for the large plot size. Finally, the PERFORMANCE-2 experiment consisted of the full bacteria-AMF-EPN combinations during the subsequent season (Table 2).

All the experiments were conducted in neighboring experimental field sites located near Prangins, Switzerland (see Table 2 for coordinates). The sites belong to Agroscope, research center of Changins, (Nyon, Switzerland) and have documented crop and management sequences for the last 30 years. The field sites chosen for the experiments had no overlapping areas to avoid cross-contaminations with inoculants. None of the experiments had irrigation systems. The soil type was sandy loam for the COMBINATION and PERFORMANCE-1 trials and loam for the PERFORMANCE-2 trial (Table 2). General agronomic preparations for all the experiments included tillage (15 cm deep) and harrowing about 4 days before seeding. The seeding machine “Hege Seedmatic” (Hege Maschinen, Eging am See, Germany) allowed the customized seeding for each plot size and arrangement (Table 2; Figure S1) and was modified to keep the seed furrows open after placing the seeds. After seeding, the plots were marked for the corresponding treatments (see Figure S1 for the exact field design of each of the three experiments) and inoculated on the same day with the beneficial soil organisms. In combination treatments, the application followed the order bacteria, EPN, and AMF.

Bacteria were applied as a cell suspension to the seed furrows (plant rows) using treatment-specific watering cans. Final cell suspensions were prepared directly on the field from bacterial stock suspensions (OD_600_ 0.15; i.e., 8 × 10^7^ CFU ml^−1^) by adjusting with water to obtain the required volumes (400 ml per meter of row) and bacterial cells (8 × 10^8^ CFU per meter of row) (Table 2). Similarly, EPN were applied in variable volumes depending on plot size using treatment-specific watering cans. They were applied to entire plots (not just the rows), and in all the cases, the final concentration was 0.5 Mio. IJs/m^2^ (equivalent to 50 IJs/cm^2^, Grewal and Peters, 2005). Finally, AMF inocula were applied manually employing 250-ml glass beakers. The material was applied directly over the seeds in the furrows, thereby gently mixing seeds and inoculum with a small hoe. AMF control plots received the same quantity of AMF-free substrate. Control plots were treated with the same volumes of BeSO-free water. Immediately after treatment application, the seeds were covered with soil using hoes to close the seed furrows. All equipment used for inoculant application was thoroughly cleaned and disinfested between manipulations using 70% ethanol.

Weed control included the application of the herbicides Azur (Omya AG, Switzerland) against monocots 2 weeks after seeding and Apell (Syngenta AGRO SA) against dicots shortly before earing (BBCH 45-50). When necessary, some persistent weeds (*Galium* spp., *Setaria* spp.) were controlled manually. No fungicides nor nematicides were applied during any of the experiments. The insecticide Karate Zeon (Lambda-Cyhalotryne, Syngenta Agro GmbH) was applied in the PERFORMANCE-2 experiment against cereal mining dipterae such as the frit fly and hessian fly conducted in 2015, but not in the 2014 COMBINATION and PERFORMANCE-1 experiments. Plots were fertilized once by supplementing nitrogen in liquid (Lonza-sol N liquid, Basel, Switzerland) at 62 kg ha^−1^ of to reach 155 units N and potassium (K_2_O) at 30.6 kg ha^−1^. The PERFORMANCE-2 trial was covered with a black hail net during the first 2–3 weeks to protect the seeds and young plants from cold conditions and predation by birds and small mammals.

### Sampling of beneficial organisms and measuring of plant traits

#### Pseudomonas bacteria

The presence of *P. protegens* CHA0-Rif and *P. chlororaphis* PCL1391-Rif was evaluated in both inoculated plots and non-inoculated control plots, as well as in the buffer zone around the experimental plots, and in the border zone (grassland) around the field site to control for possible cross contamination. This analysis was conducted four times during the growing season (i.e., at seeding, end of the winter, at earing, and maturity) in selected experiments (Table 3). For this, the root systems from four wheat plants per plot (triticale plants and grass for the buffer and border zones, respectively) were collected, pooled, washed, and gently dried with paper towels. Roots were weighed, cut into pieces (about 15 cm long), placed in 50-ml Falcon tubes (Greiner Bio One, Germany) containing 40 ml of sterile water and kept overnight at 4°C. All sampling equipment was cleaned with 70% ethanol between samples to avoid cross-contaminations. Samples were vigorously agitated on a rotary shaker at 180 rpm for 20 min, and roots were removed and dried at 80°C for 3 days to obtain the dry weight. The remaining suspensions were transferred to fresh sterile Falcon tubes on ice and centrifuged at 8,500 rpm (9,300 g) at 4°C. The supernatant was discarded and the pellet was re-suspended in 1 ml of sterile water. Each sample was then serially diluted and dilutions spread on KMB supplemented with 100 μg/ml of cycloheximide and 100 μg/ml of rifampicin (Scanferlato et al., 1990). The colonies were counted and the results were expressed as colony forming units (CFU) per gram of dry root weight.

**Table 3:** Description of the type of measurements and methods employed and timing in each of the field experiments.

**Organisms**	**Type of measurement**	**Method**	**Reference**	**COMBINATION**	**PERFORMANCE-1**	**PERFORMANCE-2**
Bacteria	Sample type	Composite samples of wheat roots	Authors	Wheat roots	Wheat roots	Wheat roots
	Quantification at seeding	CFU counting on selective media	Authors	28.03.2014	28.03.2014	27.03.2015
	Quantification during wheat growth	CFU counting on selective media	Authors	03.06.2014	03.06.2014 25.06.2014 22.07.2014	27.04.2015 18.05.2015 29.06.2015
	Tracing presence in buffer zones	CFU counting on selective media	Authors	Not done	03.06.2014	Not done
	Tracing in non-agricultural soil of border zone	CFU counting on selective media	Authors	Not done	25.06.2014 22.07.2014	Not done
AMF	Sample type	Composite samples of wheat roots	Authors	Wheat roots	Not contributed	Wheat roots
	Quantification of inoculum	Real-time qPCR using primers targeting the inocula	Authors	At harvest	Not contributed	At harvest
	Determination of AMF community	AMF community sequencing	Schlaeppi et al., 2016	At harvest	Not contributed	Not done
EPN	Sample type	Composite soil sample		12 soil cores/plot	15 soil cores/plot	15 soil cores/plot
	EPN presence: pre-inoculation (baseline)	Species-specific primers/probes and real time qPCR	Campos-Herrera et al., 2015a	27.03.2014 (Baseline)	27.03.2014 (Baseline)	27.03.2015 (Baseline)
	EPN presence: post-augmentation	Species-specific primers/probes and real time qPCR	Campos-Herrera et al., 2015a	25.06.2014	25.06.2014	17.06.2015
	EPN activity: insect-baits	*Galleria* bait	Bedding and Akhurst, 1975	25.06.2014	25.06.2014	17.06.2015
	Soil food web assemblage (nematophagous fungi, free-living nematodes and ectophoretic bacteria)	Species-specific primers/probes and real time qPCR	Atkins et al., 2005; Zhang et al., 2006; Campos-Herrera et al., 2011b, 2012, 2015b; Pathak et al., 2012	27.03.2014 (Baseline) 25.06.2014	27.03.2014 (Baseline) 25.06.2014	27.03.2015 (Baseline) 17.06.2015
Plants^a^	Height (average per plot)	Measured from shoot base to the upper growth	Authors	–	–	23.04.2015 05.06.2015 18.05.2015 01.06.2015 08.06.2015
	Weight	Eight plants	Authors	–	–	At harvest
	Density (% of plot surface covered by plants)/number of plants per linear meter	Visual scoring	Authors	–	14.05.2014 21.05.2014 11.06.2014	06.05.2015 18.05.2015 18.06.2015 29.06.2015
	Chlorophyll activity	N-tester (YARA)	Authors	–	–	08.06.2015
	Yield (g seeds/plot);	Weighing wheat seeds at dough developmental stage	Authors	–	At harvest	At harvest
	Thousand-seed weight (TSW)	Marvin seed analyzer	Gegas et al., 2010	–	–	At harvest
	Protein content (%)	Near-infrared spectroscopy	Authors	–	–	At harvest
	Insect pest and pathogen incidence	Visual counts	Authors	Weekly	Weekly	Weekly

a *Measurements were made for the three field experiments, but data are considered not representative due to the small size of the plots (COMBINATION assay) and/or the highly heterogeneous growth of the wheat plants within the plots following heavy frit fly damage in the 2014 COMBINATION and PERFORMANCE-1 assays*.

#### Arbuscular mycorrhizal fungi

The inoculation success of the different AMF inocula was traced by quantitative PCR comparing their abundance in wheat roots sampled from inoculated, non-inoculated or mock-inoculated plots (Table 3). At harvest, the root systems of four plants per plot were pooled to become one sample. The fine roots (deeper than ca. 3 cm in soil) were cut from the root system using scissors, hackled into small pieces (1–2 cm long) with a scalpel and thereby homogenizing all root fragments of the four plants. The root samples were lyophilized and then ground to a fine powder using a Retsch Ball Mill (model MM301; settings 30 s at 30 Hz using one 1-cm steel ball). DNA was extracted from ~200 mg of fine root powder utilizing the NucleoSpin® Plant II kit from Macherey-Nagel following the instructions. DNA concentrations were determined on a Varian Eclipse Fluorescence plate reader using Quant-iT PicoGreen dsDNA Assay Kit (Invitrogen) and Herring Sperm DNA (Invitrogen) as standard solution. The *R. irregulare* strains INOQ Top and SAF22 were quantified by qPCR utilizing primers developed by Alkan et al. (2006) and Bender et al. (unpublished), respectively. The AMF signals were expressed relative to a plant signal obtained with qPCR primers targeting the wheat ADP-ribosylation factor (Giménez et al., 2011). Triplicate amplifications were performed in 20 μl reactions using the HOT FIREPol® EvaGreen® qPCR Mix Plus (no ROX) from Solis Biodyne (www.sbd.ee, Estonia) on a Bio-Rad CFX96 Touch™ Real-Time PCR Detection System (www.bio-rad.com, USA). Reactions contained 4 μl qPCR mix (5X), 1 μl of each primer (10 μM), 9 μl distilled sterile water, and 5 μl template (5 ng DNA). The cycling program consisted of a 15 min initial denaturation step at 95°C followed by 40 cycles (95°C for 15 s, 63°C for 40 s for both *R. irregulare* primer sets or 60°C for 10 s for the wheat reference primers, 72°C for 20 s) and a 10 min final extension step at 72°C. Melting curve analysis consisted of a gradient from 65 to 95°C, increasing by half degrees/per 10 s to determine the uniformity of the amplicons. Raw data were imported from the qPCR cycler into the LinRegPCR program to determine the Ct and efficiency (E) values using a common fluorescence threshold for all samples (Ruijter et al., 2009). *F. mosseae* and *Claroideoglomus claroideum* were quantified with species-specific TaqMan probes following the protocols developed by Thonar et al. (2012). Template amounts were calculated for each reaction using the individual E, averaged among the replicates of each sample and expressed relative to the plant signal. Of note, for samples of the COMBINATION trial, we determined also the whole AMF community by amplicon sequencing (Schlaeppi et al., 2016).

#### Entomopathogenic nematodes, soil food web, and post-application activity

A total of 18 soil organisms were identified and quantified before application (baseline) and post-augmentation (Table 3) to detect possible trophic cascade effects due to EPN augmentation (Campos-Herrera et al., 2013). These organisms comprised seven EPN species (all previously described for the area, Campos-Herrera et al., 2015a), four free-living nematodes (FLNs) that compete with EPN for the insect cadaver (Campos-Herrera et al., 2012, 2015b), six nematophagous fungi (NF) (Campos-Herrera et al., 2015a), and one nematode surface-associated bacterium (Enright and Griffin, 2005; Campos-Herrera et al., 2011a) (Table S1). Briefly, a composite soil sample composed of several cores (2.5 cm diameter, 20 cm depth, see Table 3 for exact quantities per experiment) were taken per plot and kept on ice for transportation to the laboratory. The nematode community and other soil organisms were extracted from aliquots of 300–400 g of fresh soil by sucrose-centrifugation (Jenkins, 1964), concentrated in 1.5 ml tubes and stored at −80°C until processed, following Campos-Herrera et al. (2015a,b). Briefly, DNA was extracted from soil samples as well as from pure cultures for the generation of standard curves (when living material was available) with the Power Soil DNA Isolation Kit (MO BIO laboratories, Inc.). If no living material was available for a target organism, we employed plasmids with the whole sequence of interest to establish our positive control (Table S1; Campos-Herrera et al., 2015a). The quality and quantity of each DNA sample was analyzed prior to use (1 μl per duplicate, Nanodrop 1000, Thermo Scientific, Wilmington, DE, USA).

Species-specific primers and probes were employed in real time qPCR assessment of the 18 soil organisms (Atkins et al., 2005; Zhang et al., 2006; Campos-Herrera et al., 2011a,b, 2012, 2015a,b; Pathak et al., 2012), following the MIQE procedures (Bustin et al., 2009). All samples were run in duplicates (unknown, positive, and negative controls) employing optical 100-well gene disc reaction plates (Biolabo, scientific instruments, Switzerland) on a Corbett Research real time PCR machine. Final reactions, concentrations, and protocols were used as previously described (Campos-Herrera et al., 2015a,b). Nematode quantification from the soil samples was done with a 10-fold dilution of the DNA, whereas the identification and quantification of NF and surface-associated bacteria required the use of total DNA without dilutions (see details in Campos-Herrera et al., 2015a). A correction factor was derived from the dilution series to transform qPCR data to numbers of IJs. Finally, a sub-sample of fresh soil was dried to allow quantification per 100 g of dry soil.

In addition to the EPN soil food web, we evaluated the EPN activity at post-application sampling times (Table 3), as previously described by Campos-Herrera et al. (2015a) and Jaffuel et al. (2016). Briefly, two aliquots of 200 g of fresh soil per sample were baited with larvae of *Galleria mellonella* (Lepidoptera: Pyralidae) to test the suppressive potential of the soil. Following a modified procedure as described by Bedding and Akhurst (1975), each subsample (from augmented or not augmented plots) was baited with five final instar *G. mellonella* larvae (commercial stock, Au Pêcheur SARL Neuchâtel, Switzerland) in two independent rounds. After exposure for 4 days, the cadavers were recovered from the soil, thoroughly rinsed with tap water, and individually placed in White traps (White, 1927). Under a stereoscope, we checked for nematode emergence every 2–3 days to determine the organisms responsible for larval mortality. We recovered the nematodes in tap water upon emergence. The cadavers for which no obvious cause of death could be determined after 1 month of incubation were discarded after dissection. The DNA of the progeny leaving the cadavers was extracted using the QIAamp DNA mini kit (Qiagen), purity checked (Nanodrop system), adjusted to the range of 0.5–1 ng/μl, and species identity assessed by qPCR as described above (Campos-Herrera et al., 2015a; Jaffuel et al., 2016).

#### Plant traits

A total of eight measurements recorded the evolution of plant growth, productivity, and health. They were: average plant height per plot, plant density per plot, chlorophyll activity (N-tester), seed yield, thousand-seed weight, plant weight, plant protein content, and presence of pest insects and pathogens (Table 3). Regular monitoring of the experiments ensured the status of development into each phenostage. Most of the agronomical traits presented herein were measured at harvest (Table 3).

### Statistical analysis

All experimental field trials presented a Randomized Complete Block design (Figure S1). The data from each group of beneficial organisms were analyzed following standard procedures for their data presentation, transformation, standardization, and normalization whenever necessary. In the case of the EPN activity, data from the *G. mellonella* baits were expressed as the percentage of larval mortality per plot, averaged by treatment. The activity was determined with respect to the total mortality caused only by nematodes. For the EPN soil food web analysis, all the organisms (EPN, FLN, NF, and bacteria) quantified by using qPCR were expressed per 100 g of dry soil. The parasitism of nematodes by NF was expressed as “infection rate” (IR), which was calculated by dividing the DNA quantity of each species by the total amount of DNA (Campos-Herrera et al., 2012; Duncan et al., 2013). Similarly, to estimate the total FLN and NF, we divided all data within a species by the highest measurement for that species, which allowed the standardization of the units of measurement among species ranging from 0 to 1 (de Rooij-van der Goes et al., 1995).

Unless specified, all significant differences between treatments were assessed by one–way ANOVA, using Tukey's HSD test, considering block as co-variable (V 20.0, IBM SPSS Inc., Chicago, IL). In some cases, *t*-tests were employed to compare pre- and post-augmentation or control vs. a specific treatment. If necessary, data were transformed to conform the assumptions of normality and equal variances (transformation method is indicated with the respective statistics). The bacterial colonization data were statistically assessed with a non-parametric Kruskal-Wallis test, followed by a *post-hoc* test (Dunn's test). With the exception of the *Pseudomonas* root colonization data (presented as log_10_ of the obtained values ± SEM) all data are presented as mean ± SEM of untransformed values.

## Results

### Survival and persistence of *pseudomonas* inoculants

**Figure 1:**
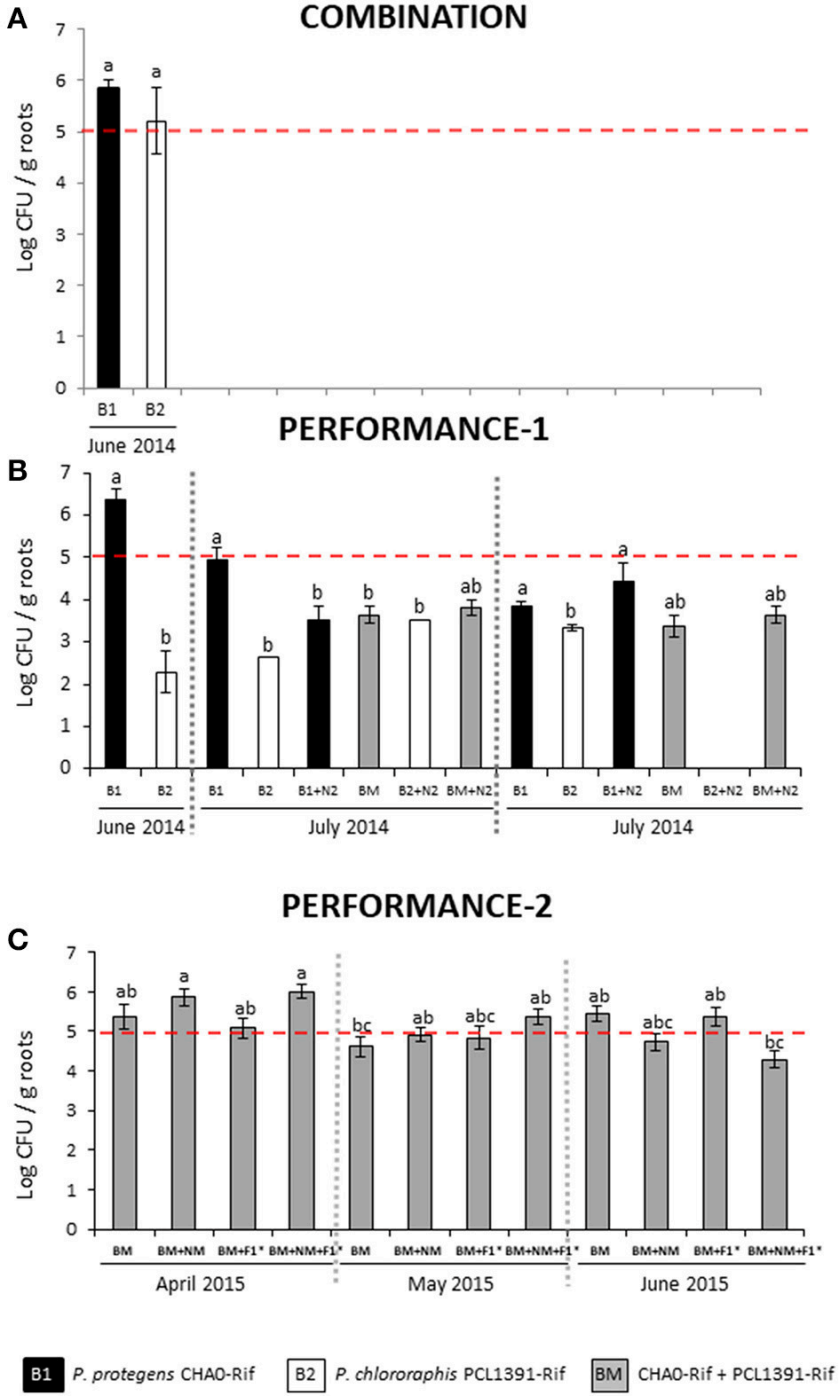
Survival of *Pseudomonas protegens* strain CHA0-Rif (B1) and *Pseudomonas chlororaphis* strain PCL1391-Rif (B2) on wheat roots in the COMBINATION **(A)**, PERFORMANCE-1 **(B)**, and PERFORMANCE-2 **(C)** field trials. Bacterial strains were inoculated individually or in combinations with the entomopathogenic nematode (EPN) *Heterorhabditis bacteriophora* (N2), an EPN mixture (NM; comprising *Heterorhabditis megidis, H. bacteriophora*, and *Steinernema feltiae*) and the arbuscular mycorrhizal fungus *Rhizoglomus irregularis* (F1*). Inoculants were monitored by selective plating on KMB supplemented with rifampicin (100 μg/ml) and cycloheximide (100 μg/ml) at three different time points following seed furrow inoculation. The dashed red line indicates the generally agreed threshold (~10^5^ CFU per g root) required to provoke beneficial plant effects with plant growth-promoting pseudomonads (Haas and Défago, 2005). Bar graphs show means of log_10_ transformed CFU values per gram of dry roots weight (± SEM). Significant differences between treatments were calculated with one-way ANOVA (significance level *P* < 0.05) followed by the Tukey *post-hoc* test, or with a non-parametric Kruskal-Wallis test (significance level *P* < 0.05), followed by Dunn's test for *post-hoc* comparisons. Different letters indicate statistical significance at *P* < 0.05. Inoculants were not detected in the buffer and border zones of the field assays. No Rifampicin-resistant background population was detected at the field sites.

In the COMBINATION trial, *P. protegens* CHA0-Rif and *P. chlororaphis* PCL1391 reached similar population densities that surpassed the threshold of ~10^5^ CFU per gram of roots, which is the level needed for a plant-beneficial effect (Haas and Défago, 2005) (Figure 1A; Table 4). However, in the PERFORMANCE-1 trial, the population density of the *P. chlororaphis* strain on wheat roots was significantly lower (*P* < 0.05) than the density of the *P. protegens* strain at all three monitoring times (Figure 1B; Table 4). In this trial, in general, for strain CHAO-Rif, alone or in the combinations, we observed a better progression of the population if compared with the strain PCL1391-Rif. If both strains were present in the same treatment, our agar plates almost only reported *P. protegens* CHA0-Rif colonies (personal observation). Moreover, in clear contrast to CHA0-Rif, PCL1391-Rif never approached the population threshold for plant-beneficial effects, neither when applied alone nor when combined with other BeSO, (Figure 1B; Table 4). In combination with a commercial population of the EPN *H. bacteriophora*, the density of CHA0-Rif was significant reduced for the June 2014 sample. July 2014, i.e., about 1 month later, CHA0-Rif still maintained its population density in presence of the nematode inoculant while PCL1391-Rif was no longer detectable (Figure 1B; Table 4). In the 2015 PERFORMANCE-2 trial, bacterial numbers approached or surpassed the threshold for plant-beneficial effects at all three sampling times (Figure 1C; Table 4). In general, no significant differences among treatments were observed, but there was a trend of higher bacterial population densities in the April 2015 and May 2015 samplings when bacterial inoculants where combined with the EPN inoculant mixture. In contrast, an opposite trend was observed for June 2015 samples. Finally, as already observed in the PERFORMANCE-1 trial, strain *P. protegens* CHA0-Rif dominated the colonization, while *P. chlororaphis* PCL1391 was hardly detected (Figure S2).

**Table 4:** Statistical analysis for beneficial soil organisms and plant traits in the three field experiments.

**Organisms**	**Type of measurement**	**Statistical method**	**COMBINATION^a^**	**PERFORMANCE-1^a^**	**PERFORMANCE-2^a^**
Bacteria	CFU quantification I	One–way ANOVA (Tukey's HSD test); Kruskal-Wallis (Dunn's test)	*F*_(1, 6)_ = 0.1526, n.s	*F*_(1, 2)_ = 2.571, ^**^	*F*_(3, 15)_ = 0.991, n.s.
	CFU quantification II	One–way ANOVA (Tukey's HSD test); Kruskal-Wallis (Dunn's test)	–^b^	*F*_(5, 12)_ = 3.675, ^**^	*F*_(3, 16)_ = 0.656, n.s.
	CFU quantification III	One–way ANOVA (Tukey's HSD test); Kruskal-Wallis (Dunn's test)	–	*F*_(4, 15)_ = 2.311, n.s.	*F*_(3, 16)_ = 1.570, n.s.
AMF	Quantification of INOQ Top^c^	One–way ANOVA	*F*_(2, 9)_ = 10.42, ^**^	Not contributed	–
	Quantification of SAF22^c^	*T*-tests	*T* = 10.377 ^***^	Not contributed	–
	Quantification of INOQ Top in combination samples^c^	One–way ANOVA	*F*_(5, 18)_ = 3.712, ^*^	Not contributed	*F*_(4, 36)_ = 0.571, n.s.
EPN	EPN presence: pre-inoculation (baseline)	One–way ANOVA (Tukey's HSD test)	*F*_(7, 24)_ = 1.273, n.s.	*F*_(6, 21)_ = 0.525, n.s.	*F*_(4, 40)_ = 0.281, n.s.
	EPN presence: post-augmentation	One–way ANOVA (Tukey's HSD test)	*F*_(7, 24)_ = 4.604, ^**^	*F*_(6, 21)_ = 2.194, ^§^	*F*_(6, 21)_ = 2.888, ^*^
	EPN activity: insect-baits	One–way ANOVA (Tukey's HSD test)	*F*_(7, 24)_ = 3.317, ^*^	*F*_(6, 21)_ = 1.243, n.s.	*F*_(6, 21)_ = 0.722, n.s.
	Free-living nematodes: pre-inoculation	One–way ANOVA (Tukey's HSD test)	*F*_(7, 24)_ = 1.051, n.s.	*F*_(6, 21)_ = 0.498, n.s.	*F*_(6, 21)_ = 0.119, n.s.
	Free-living nematodes: post-augmentation	One–way ANOVA (Tukey's HSD test)	*F*_(7, 24)_ = 0.395, n.s.	*F*_(6, 21)_ = 1.025, n.s.	*F*_(6, 21)_ = 0.341, n.s.
	Nematophagous fungi: pre-inoculation	One–way ANOVA (Tukey's HSD test)	*F*_(7, 24)_ = 0.675, n.s.	*F*_(6, 21)_ = 0.288, n.s.	*F*_(6, 21)_ = 0.618, n.s.
	Nematophagous fungi: post-augmentation	One–way ANOVA (Tukey's HSD test)	*F*_(7, 24)_ = 5.820, n.s.	*F*_(6, 21)_ = 1.384, n.s.	*F*_(6, 21)_ = 0.582, n.s.
Plants	Height (at harvest)	One–way ANOVA (Tukey's HSD test)	–	–	*F*_(8, 72)_ = 1.009, n.s.
	Density (% plot covered by plants at harvest)	One–way ANOVA (Tukey's HSD test) Kruskal-Wallis (Dunn's test)	–	*F*_(7, 88)_ = 17.219, ^***^	*F*_(8, 72)_ = 0.756, n.s.
	Chlorophyll activity (N-tester)	One–way ANOVA (Tukey's HSD test)	–	–	*F*_(8, 72)_ = 0.161, n.s.
	Yield (g seeds/plot)	One–way ANOVA (Tukey's HSD test)	–	*F*_(7, 23)_ = 2.069, ^**^	*F*_(8, 72)_ = 0.026, n.s.
	Thousand-seed weight (TSW)	One–way ANOVA (Tukey's HSD test)	–	–	*F*_(8, 72)_ = 0.129, n.s.
	Protein content	One–way ANOVA (Tukey's HSD test)	–	–	*F*_(8, 72)_ = 0.300, n.s.

a Data are presented as the statistical values, degree of freedom and probability levels:

§ P < 0.1,

* P < 0.05,

** P < 0.01,

*** *P < 0.001, n.s., not significant*.

b *For these variables, obtained data were not representative because of highly heterogeneous growth of the wheat plants within the plots following frit fly damage and thus were not considered for statistical analysis*.

c *Statistics corresponding to data from two sets of primers, i.e., by Alkan et al. (2006) for INOQ Top, and Bender et al. (unpublished) for SAF22*.

In all three field trials, no rifampicin-resistant bacteria were detected in the non-inoculated control treatments, in the buffer zones or in the grassland border zones at the experimental sites (data not shown), hence, the reported CFU data for the augmented bacteria required no baseline correction.

In general, the applied bacteria survived under field conditions until the end of the crop season (Figure 1), although the threshold required to provoke beneficial plant effects (~10^5^ CFE per g root) was not always attained in all the trials or treatments. Inoculation was successful in all, with good traceability of the different bacterial inocula without cross-contamination. Consistently, the *P. protegens* inoculant showed higher presence on wheat roots, but the effects of the combination with other BeSO were not conclusive and depended on the BeSO species, on the time of exposure to the field conditions and differed between trials.

### AMF inoculation success

**Figure 2:**
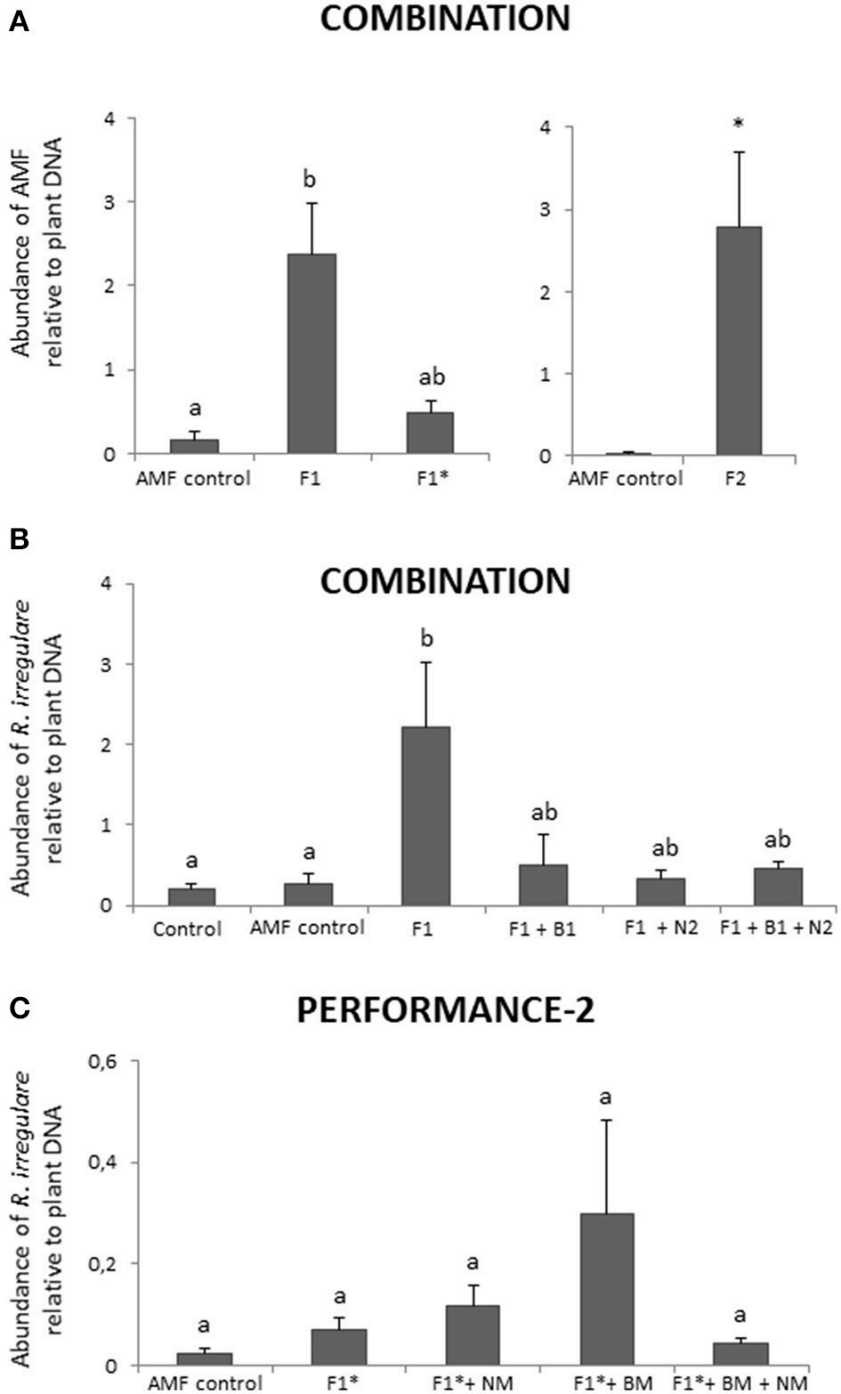
Abundance of *Rhizoglomus irregulare* in wheat roots in the COMBINATION **(A,B)** and PERFORMANCE-2 **(C)** field trials. **(A)** In the COMBINATION experiment, *R. irregulare* strain INOQ TOP was inoculated comparing high (F1) vs. low (F1*) dosages, with one of the treatments including the AMF strain SAF22 (F2). **(B)** In the same experiment, *R. irregulare* INOQ TOP (F1) was quantified in combination with bacteria, i.e., *Pseudomonas protegens* CHA0-Rif (B1), and nematodes, i.e., *Heterorhabditis bacteriophora* Andermatt (N2). **(C)** In the PERFORMANCE-2 experiment, *R. irregulare* INOQ TOP at the lower dosage (F1*) was used for the combination treatments with bacterial mixture (BM; i.e., *P. protegens* + *Pseudomonas chlororaphis*) and nematode mixture (NM; i.e., *Heterorhabditis megidis* + *H. bacteriophora* + *Steinernema feltiae*; for details see Figure S1). Control, non-inoculated control; AMF control, substrate control for AMF inoculation. *R. irregulare* was measured with quantitative PCR employing species-specific primers developed by Alkan et al. (2006) for INOQ TOP or their modified variants with enhanced specificity for SAF22 (Bender et al., unpublished). Bar graphs report mean normalized (*R. irregulare* relative to plant DNA) abundance (± SEM; COMBINATION, *n* = 4; PERFORMANCE-2, *n* = 7–9). Statistical analyses were performed on log-transformed data; asterisks and different letters indicate statistical significance at *P* < 0.05 for *t*-test and one-way ANOVA followed by the Tukey *post-hoc* test, respectively.

For the COMBINATION trial we mainly used the locally well-adapted AMF *R. irregulare* (Schlaeppi et al., 2016) (Table 1). We confirmed successful wheat root inoculation for both *R. irregulare* strains that we tested, as well as our custom strain SAF22 and the commercial inoculum INOQ Top (Figure 2A; Table 4). The higher dosage of the inoculum INOQ Top (80 g per row) corresponded approximately to the amount of SAF22 inoculum and it appeared that both *R. irregulare* strains colonized the wheat roots to a similar extent. The reduced dosage of the commercial inoculum (16 g per row) was reflected in lower levels of root colonization and only showed a minor tendency of augmentation. We also traced the inoculation of the *F. mosseae* strain SAF11 and the *C. claroideum* strain SAF12 using specific qPCR primers (Thonar et al., 2012). We did not detect these AMF species at the field site (data not shown) confirming the findings of a previous AMF community profiling (Schlaeppi et al., 2016). Hence, we concluded that these strains failed to establish at the tested field site in the wheat roots. In summary, *R. irregulare* could be augmented in wheat AMF communities using the strains SAF22 or INOQ Top, while this was not successful for *F. mosseae* SAF11 and *C. claroideum* SAF12.

Plots in which soil beneficial organisms showed low AMF colonization levels were not different from control (nothing applied) and mock (application of carrier substrate without AM fungus) plots (Figure 2B; Table 4). These measured abundances of *R. irregulare* correspond to the native strain in the field and indicated that the application of the carrier substrate on its own did not affect the root colonization by the AM fungus. Although, the level of root colonization by *R. irregulare* showed a slight tendency to increase in the combination treatments of the AM fungus with bacteria, nematodes or both, the AM fungus was not augmented to the same extent as in single application. These first insights on combining soil beneficial organisms suggest possible negative effects on the AMF inoculum if combined with bacteria or nematodes.

In the PERFORMANCE-2 experiment, the commercial *R. irregulare* strain INOQ Top was inoculated using a lower dosage level to larger plots compared to the previous experiments (Table 2). Again, there was a tendency of increased colonization of the wheat roots in the combined treatments, however, high inter-plot variation precluded statistic support for this effect (Figure 2C; Table 4). It remains to be validated whether the colonization by this *R. irregulare* strain is particularly facilitated if applied in combination with the *Pseudomonas* bacteria.

In summary, *R. irregulare* successfully colonized wheat roots if inoculated alone, and in the combination experiments, we found varying augmentation efficiencies for *R. irregulare* if combined with pseudomonads, EPN or both, indicating that interactions with these beneficial soil organisms are context dependent.

### Nematode survival, activity, and interactions with soil food web members

In all plots, very low numbers of background populations were detected as also found by Campos-Herrera et al. (2015a) and Jaffuel et al. (2017) in the same area. Five to seven species naturally occurred at the experimental field sites, and these species included the taxa that we augmented. Prior to inoculations (baseline; Table 3), there were no differences among treatments for any measured variable (EPN, free-living nematodes and nematophagous fungi; data not shown) in any of the three field trials. The evaluation of EPN soil food web members (free-living nematodes and nematophagous fungi) only revealed natural temporal fluctuations between baseline (pre-inoculation) and post EPN augmentation (data not shown), whereas their presence was not significantly affected by the EPN augmentation (alone or in combination) (Figure S3; Table 4). The nematophagous fungi and free-living nematodes species were in agreement with those already described by Campos-Herrera et al. (2015a,b) and Jaffuel et al. (2017). Finally, the ectophoretic bacterium *P. nematophilus* was not detected in any of plots (control or augmented).

**Figure 3:**
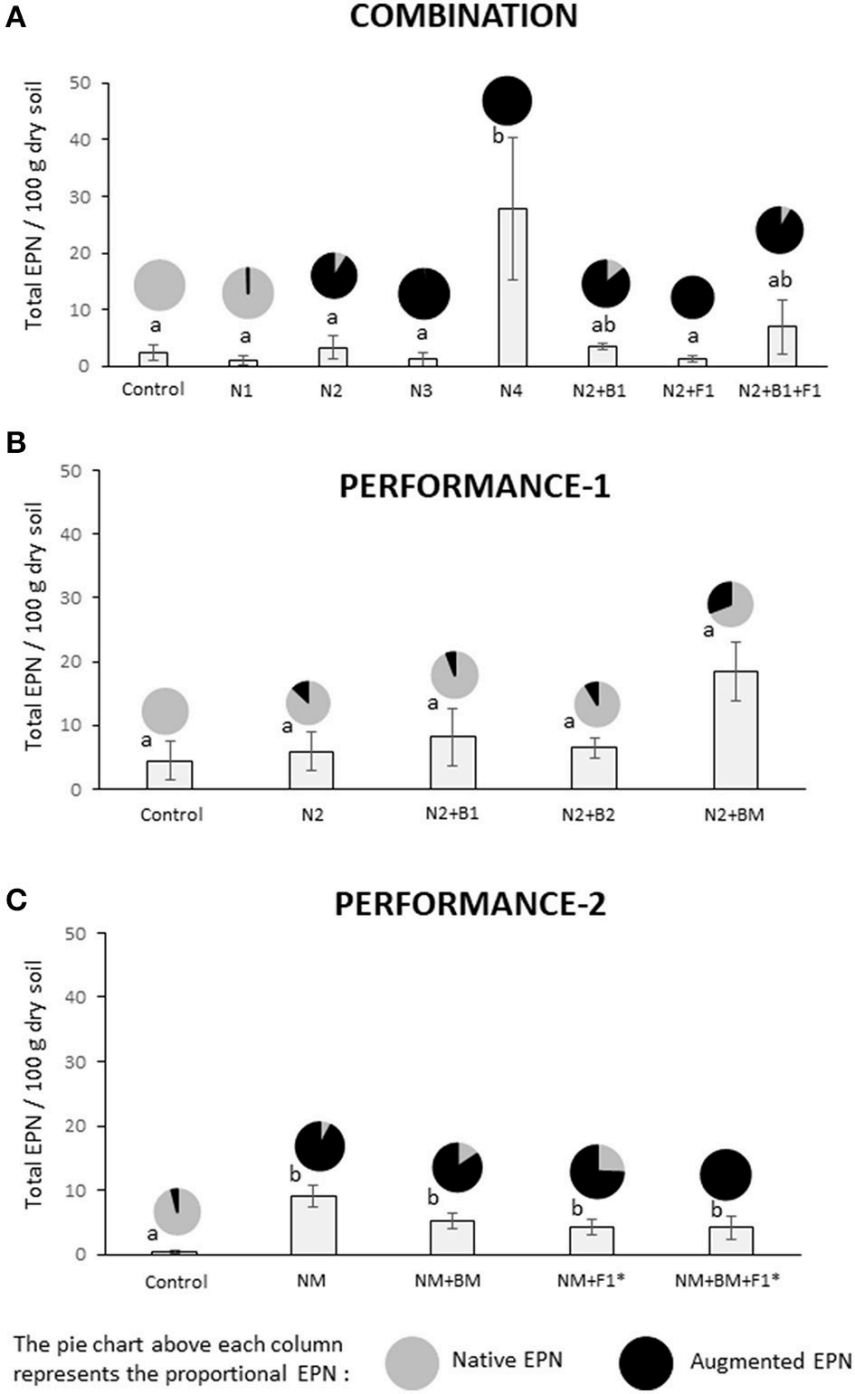
End of the season presence of inoculant and resident entomopathogenic nematodes in the COMBINATION **(A)**, PERFORMANCE-1 **(B)**, and PERFORMANCE-2 **(C)** field trials. Four different EPN species *Heterorhabditis megidis* (N1), *Heterorhabditis bacteriphora* (N2), *Steinernema carpocapsae* (N3), and *Steinernema feltiae* (N4) were inoculated individually or in combination with *Pseudomonas protegens* (B1), *Pseudomonas chlororaphis* (B2) and *Rhizoglomus irregularis* at two dosages (F1 and F1*). Mixtures of EPN (N1+N2+N4) or of the two bacteria (B1+B2) are indicated with NM and BM, respectively (for details see Figure S1). To determine the persistence of the EPN in soil of the different nematode inoculants as well as the impact of each treatment on the resident population of entompathogenic nematodes (EPN), a DNA extraction procedure followed by a qPCR approach was performed. Data are expressed as total EPN 100 g^−1^ of dry soil. Bar graphs report means (± SEM) and pie-charts show the proportion of native EPN vs. augmented EPN. Significant differences between treatments were calculated with one-way ANOVA (significance level *P* < 0.05) followed by the Tukey *post-hoc* test. Different letters indicate statistical significance at *P* < 0.05.

**Figure 4:**
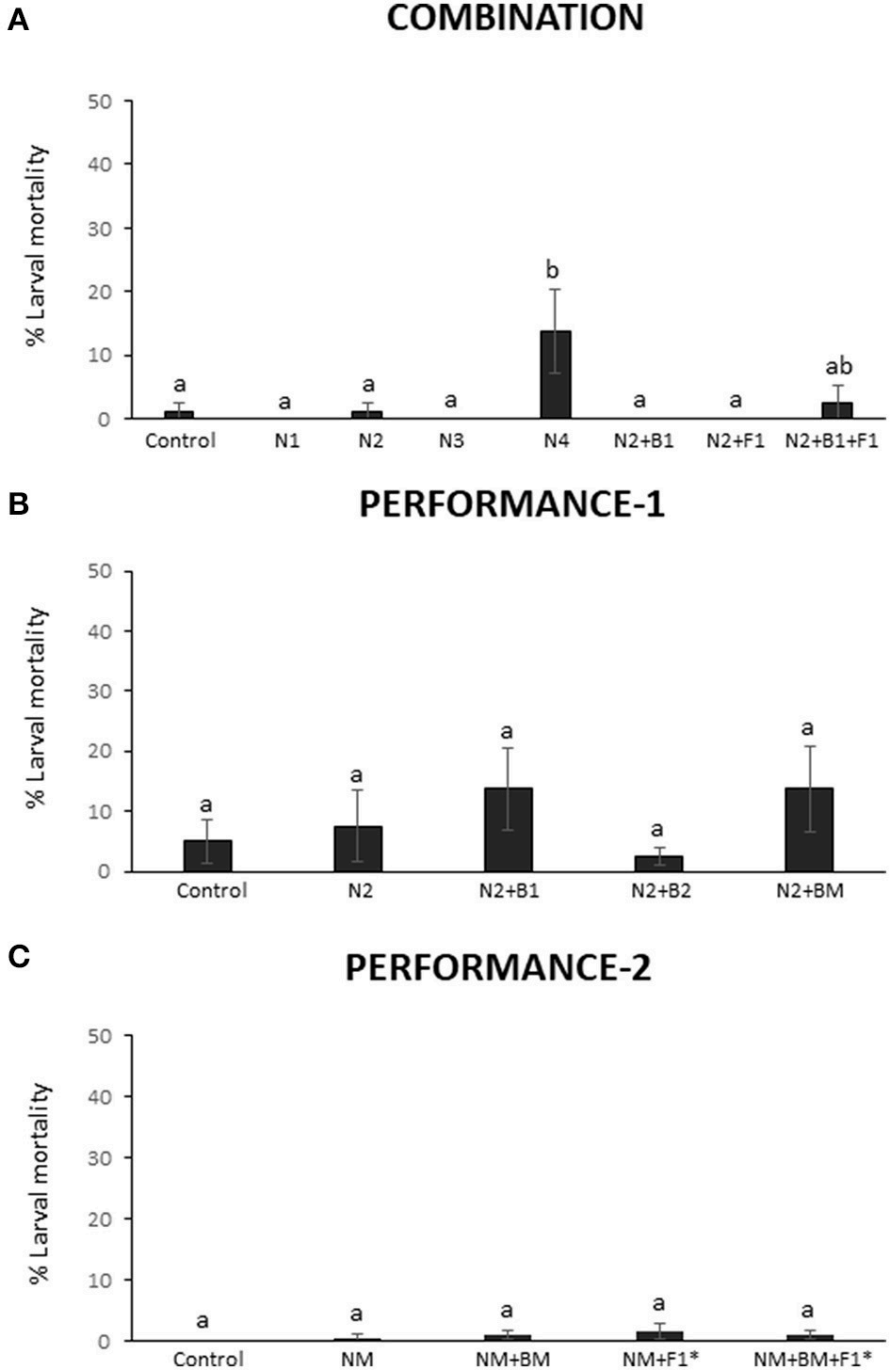
Activity of entomopathogenic nematodes (EPN) post application in three field trials. EPN activity was quantified by a *Galleria mellonella* larvae infection assay in soil samples from the **(A)** COMBINATION, **(B)** PERFORMANCE-1 and **(C)** PERFORMANCE-2 trials. Inoculants were *Heterorhabditis megidis* (N1), *Heterorhabditis bacteriophora* (N2), *Steinernema carpocapsae* (N3), and *Steinernema feltiae* (N4), individually or in combination with *Pseudomonas protegens* (B1), *Pseudomonas chlororaphis* (B2), and *Rhizoglomus irregularis* at two dosages (F1 and F1*). Mixtures of EPN or bacteria are indicated with NM and BM, respectively (for details see Figure S1). Bar graphs report means (± SEM). Significant differences between treatments were calculated with one-way ANOVA (significance level *P* < 0.05) followed by the Tukey *post-hoc* test. Different letters indicate statistical significance at *P* < 0.05.

In the COMBINATION trial, the EPN species *H. megidis* and *S. carpocapsae* were recovered in only 25% of the plots, 4 months after augmentation. In contrast, the species *S. feltiae* and *H. bacteriphora*, of which the latter was also combined with other BeSO, were detected in 100% of the plots, at the end of the season. The augmentation with *S. feltiae* was the only treatment with a significant increase in total EPN numbers compared with the native populations (Figure 3A; Table 4). The remarkable persistence of *S. feltiae*, which was the only species detected in the soil in their corresponding plots, was in agreement with the nematode activity measured in the laboratory as % mortality of *G. mellonella* producing progeny. This was the only treatment with significantly higher activity than the control in the whole trial (Figure 4A; Table 4).

In the PERFORMANCE-1 trial, we only augmented certain plots with *H. bacteriophora*. This EPN was detected in about 50% of the plots when applied alone or in combination with the bacterial inoculant *P. chlororaphis* and in about 75% of the plots when applied with the *P. protegens* in different combinations. No significant difference in EPN populations (qPCR measurements) and their activity (% larval mortality) was observed between plots where the EPN were applied alone or in combination with bacterial inoculants. Nor were they different from control plots (Figures 3B, 4B; Table 4). In both trials, COMBINATION and PERFORMANCE-1, there was a slight trend to detect more *H. bacteriophora* in the combined treatment with AMF and/or bacterial inoculants (N2+B1+F1 and N2+BM, respectively) (Figures 3A,B). The same trend was also observed for nematode activity (Figures 4A,B). In the PERFORMANCE-1 field trials, *Steinernema affine* (Table S1) was the dominant native EPN species in the soil of the experimental plots as determined by qPCR (Figure 3B).

In the PERFORMANCE-2 trial, the augmented EPN species (a mix of *S. feltiae, H. megidis*, and *H. bacteriophora*) could be detected in 100% of the plots inoculated with the three EPN, alone or in combination with the *Pseudomonas* inoculants, in 91% of the plots where they were combined with AMF and in only 44.4% of the plots when combined with both bacterial and AMF inoculants. Again, the species *S. affine* was dominant among the native taxa as displayed in the proportional chart, although, contrary to the PERFORMANCE-1 trial, native species were largely displaced in all the treatments where EPN were applied (Figure 3C). All plots with EPN application showed significantly higher total numbers of EPN than the control plots (Figure 3C; Table 4). All the augmented EPN species were detected in each of the plots, but *S. feltiae* and *H. bacteriophora* dominated. The nematode activity was low and did not significantly vary among treatments (Figure 4C; Table 4). The progeny from the activity tests belonged mainly to *H. bacteriophora* (62.5%), followed by *S. feltiae* (34.5%), in all the cases we found mixed EPN-free-living nematodes emergence as observed in previous studies in Swiss soils (Jaffuel et al., 2016, 2017).

In general, inoculated EPN persisted during the crop season and remained active until the time for wheat harvest, but with limited pest suppressive potential as measured with a *Galleria* larvae infection assay. We observed that EPN application increased the total numbers of EPN only in specific treatments, displacing at least partially the native populations (Figure 3). No long-term effect was observed with respect to soil organisms that can be expected to be modulated by EPN augmentation, such as nematophagous fungi and free-living nematodes. The combined application of EPN with other BeSO indicated compatibility with respect to their persistence, prevalence, and activity, when compared with the single EPN application, but some differences depending on EPN species and co-inoculant identity were observed. As for the bacterial and AMF inoculants, the success of EPN inoculants appeared to be context dependent.

### Agronomic impact of the applied beneficial soil organisms

**Figure 5:**
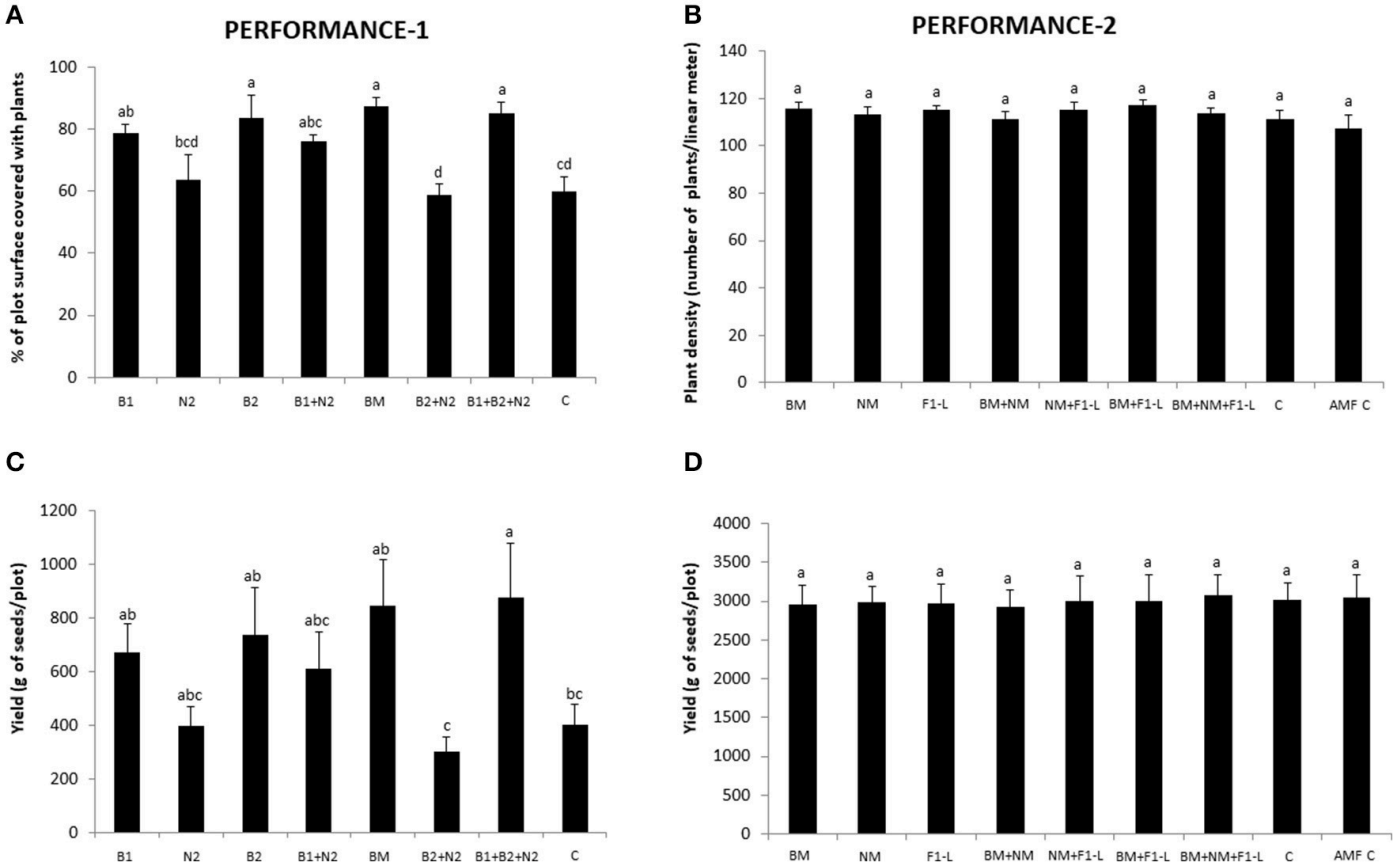
Impact of field inoculations with beneficial organisms on plant performance in the PERFORMANCE-1 **(A,C)** and PERFORMANCE-2 **(B,D)** trials. Plant performance was evaluated in terms of plant density **(A,B)** and yield (weight of wheat seeds) **(C,D)** for each plot. The PERFORMANCE-1 experiment was exposed to heavy natural infestation with the firt fly (*Oscinella frit*) causing significant plant damage. Plant density in the PERFORMANCE-1 trial was therefore determined by visual scoring the percentage of plot area covered by wheat plants in this experiment while it was determined by counting the number of plants per linear meter in the PERFORMANCE-2 experiment, which had no measurable frit fly damage. Inoculants were *Pseudomonas protegens* (B1), *Pseudomonas chlororaphis* (B2), individually or in combination with *Heterorhabditis bacteriophora* (N2) and *Rhizoglomus irregularis* (F1*). Mixtures of the two bacteria or of the entomopathogenic nematodes (*Heterorhabditis megidis, Heterorhabditis bacteriophora*, and *Steinernema feltiae*) are indicated with NM and BM, respectively (for details see Figure S1). C, non-inoculated control; AMF-C, substrate control for AMF inoculation. Bar graphs report means (± SEM). Significant differences between treatments were calculated with one-way ANOVA (significance level *P* < 0.05) followed by the Tukey *post-hoc* test. Different letters indicate statistical significance at *P* < 0.05.

The 2014 trials (COMBINATION and PERFORMANCE-1) were intentionally not subjected to standard pesticide treatments and suffered from heavy attack by frit flies (*Oscinella frit*). For the small scale COMBINATION trial, insect damage was very patchy and therefore not agronomically representative and not included in the plant performance analyses. The larger plot sizes in the PERFORMANCE-1 trial permitted analysis of agronomically relevant plant density and seed yield data (Table 4). The % of plot surface covered with plants was significantly higher in augmentation plots than in the control treatment when the two bacterial inoculants, *P. protegens* and *P. chlororaphis*, were applied individually (treatments B1 and B2, respectively) or as a mixture with and without the EPN (treatments BM and B1+B2+N2, respectively) (Figure 5A). Seed yield per plot followed a similar pattern, but only the combined treatment with both bacterial strains and the EPN showed significantly higher values than the control (Figure 5C). AMF effects could not be examined in the PERFORMANCE-1 trial due to limited inoculum production. Nevertheless, the neighboring COMBINATION experiment indicated that seedling survival after frit fly attack tended to be higher in plots inoculated with *R. irregulare* (Figure S4). In the 2015 PERFORMANCE-2 trial, plots were subjected to pesticide treatment, no pest damage was observed and all plant traits were considered in the analysis. However, none of these measures, including plant density and seed yield per plot (Figures 5B,D) nor the other plant performance traits (Figure S5; Tables 3, 4) differed significantly from the control treatment.

In summary, when wheat was exposed to biotic stress (i.e., a heavy insect pest attack in 2014) a significant positive effect of the application of BeSO, notably *Pseudomonas* bacteria, on performance of the crop was observed. The presence of the EPN was only beneficial when combined with both bacteria together. In absence of a biotic stress conditions, as in the PERFORMANCE-2 trial in 2015, there was no measurable plant-beneficial effect of the presence of BeSO, highlighting the context dependence of their protective effect on the crops.

## Discussion

Overall, the three field experiments showed consistent results: (1) the inoculated BeSO persisted until the end of the crop season, although their prevalence gradually declined with time; (2) in most of cases, the introduced BeSO in augmented plots were consistently present at higher levels than the native populations, without cross-contamination between plots; (3) the augmented BeSO integrated with or displaced the natural community to varying degrees depending on the strain/population and dosage; and (4) the combined application of *Pseudomonas*, EPN, and AMF showed only beneficial effects under conditions with an insect outbreak. In particular and contrary to our expectations, our current tripartite BeSO inoculant system (bacteria + EPN + AMF) did not provide clear additive or synergistic positive effects to allow a better performance of wheat than the application of the individual BeSO. Overall, our results are in agreement with the previous observation that the combination of various BeSO can lead to a beneficial effect under certain conditions (Frey-Klett et al., 2007; Ansari et al., 2010; Walker et al., 2011; Couillerot et al., 2012), but mainly have similar effects as single applications (Tarasco et al., 2011; Glare, Hurst, and Narciso, personal communication). We can conclude that there is still a large gap between the promising results from BeSO applications under controlled experiments (laboratory and greenhouse settings) and their performance under field conditions.

Many factors can explain this difference between applications in laboratory/greenhouse and field settings. The characteristics of a particular agroecosystem (i.e., soil type, soil geochemistry, humidity, plant genotype, climate, etc.) play a decisive role in determining the success of augmented BeSO. From a biogeographic point of view, the selection of the BeSO should take in consideration the biology and ecology of the BeSO. The soil and environmental conditions in the target soils should match the conditions within the range of the natural occurrence of the BeSO, in order to obtain the desired activity. The soil is a complex medium, with physicochemical and biological interactions that vary over time and space (Ritz and van der Putten, 2012). In the three trial, the general characteristics of the soil were largely similar (Table 2), although unnoticed microhabitat differences might patchily occur and produce internal stochasticity, a factor that is better controlled in any greenhouse experiment where often soils are homogenize first and treatments are confined to smaller experimental units such as pots. In a field experiment, fundamental differences in soil chemistry (acid soils vs. basic soils, presence of micronutrients, etc.) and soil physical properties (texture, pore size, compaction, available water, etc.), should be considered to select the most appropriate BeSO (Schlaeppi and Bulgarelli, 2015). For example, AMF mostly perform better in low nutrient soils (Pellegrino et al., 2012, 2015). Also the effects of AMF on crop productivity are highly dependent on the plant species or genotypes investigated (Lekberg and Koide, 2005): plants and crops with fine roots such as wheat (as in this study) are usually less responsive to AMF compared to species with thicker roots such as red clover (Köhl et al., 2016). Similarly, EPN species have ecological and habitat preferences that are largely determined by texture and moisture of soils (Campos-Herrera et al., 2013, 2016; El-Borai et al., 2016). Soil physico-chemical characteristics can also impact persistence and activity of *Pseudomonas* species (Natsch et al., 1996; Troxler et al., 2012; Mascher et al., 2014; Imperiali et al., 2017). Hence, locally adapted species might have an advantage in persistence over exotic organisms that are not present in the target soil (Schlaeppi et al., 2016).

In addition to the abiotic soil conditions, BeSO inoculants are also subjected to interactions with the resident soil organism community. The diversity of soil organisms can contribute to buffering, masking and silencing beneficial effects of inoculations. Again, this is a major difference with controlled experiments in the growth chamber or greenhouse where conditions usually limit or simplify the interactions of inoculant BeSO with the naturally present soil organisms and the target crop. Often laboratory or greenhouse experiments are conducted with sterilized soils, with entirely or greatly reduced abundance of native soil organisms. Under field conditions, there are also spatial and temporal differences in these effects on the augmented BeSO. This is particular relevant when considering naturally occurring populations of the BeSO. In our experiments, we observed that the native populations of AMF and EPN were displaced to varying degrees, depending on the BeSO species/population inoculated in the field plots. In agreement with Schlaeppi et al. (2016) and Jaffuel et al. (2017), we also observed that augmented BeSO species that also occurred naturally in the area performed better than those that were not represented or only at low numbers. The fact that virtually no cross-contaminations with inoculants occurred between plots and in many cases the displacement of native populations was corrected by the time of harvest, i.e., returning to the original numbers/presence of native populations, underscore that these introductions have only low and transient impacts on the native populations. Yet, more studies are needed to evaluate the potential long-term impacts of implementing inoculation strategies of single or combined BeSO, especially if inoculants are not native or no present in the area of application (Abate et al., 2017; Hardt et al., 2017).

Here we introduce a comprehensive toolbox to trace Pseudomonads, AMF, and EPN after application. Some of the BeSO did not reach the numbers known to be required to reach beneficial plant effects (Haas and Défago, 2005), did not persist well-after application (i.e., the EPN species *H. megidis* and *S. carpocapsae*), or did not establish following field inoculation (i.e., the AMF species *F. mosseae* and *C. claroideum*). Nevertheless, results for some isolates and combinations were highly promising. Under the experimental field settings, the bacterium *P. protegens* CHA0, the AMF *R. irregularis* and the EPN *S. feltiae* established very well. Under conditions with high biotic stress (frit fly infestation in the PERFORMANCE-1 trial), the combination of bacterial and EPN inoculants produced the highest yields. Because such ecological conditions will change from one season to another, the development of a pre-application diagnosis tool may help the choice of an optimized BeSO (Schlaeppi and Bulgarelli, 2015; Schlaeppi et al., 2016). For example, areas strongly impacted by plant diseases and pests might benefit from the integration of various *Pseudomonas* bacteria. Whereas, the presence of insect pests will better support the development and persistence of native and augmented EPN, thereby enhancing their protective effects. Finally, selecting BeOS, in particular AMF, that are compatible with local soil conditions (e.g. low or high nutrient content) is highly advisable (Pellegrino et al., 2015; Schlaeppi et al., 2016).

Advancing our understanding of the soil-plant interface in its broadest sense is critical to achieve sustainable agriculture (Adl, 2016). We evaluated the simultaneous application of three types of BeSO (bacteria, EPN, and AMF) and its impact on wheat productivity under realistic field conditions. While we confirmed the prevalence and persistence of the three organisms throughout the season, their beneficial effects were variable and differed between inoculant strains. Clear beneficial effects on wheat growth were observed only when the plants were exposed to high insect infestation. We learned that there is still a major gap in our understanding of the capacities of BeSO to enhance plant performance under well-controlled conditions and their performance and impacts when applied to the field. We believe that to close this gap and for the successful use of BeSO in agroecosystems there is an urgent need to unravel the context dependency of effective BeSO augmentations. Optimizations should go toward adapting and fine-tuning the selection of inoculant strains that are well-adapted to local abiotic and biotic soil conditions. Advancing such an integrative and context-dependent approach is vital before next-generation, sustainable agriculture, in which field crops are protected by applying beneficial soil organisms instead by agrochemicals becomes imaginable.

## Author contributions

KS, MvdH, MM, FM, TT, CK, and RCH planned the experiments and supervised the study. NI, XC, KS, GJ, SB, FD, MF, RBP, DV, MvdH, MM, FM, CK, and RCH contributed in the field experiments and collected the data. NI, XC, KS, MF, CK, and RCH analyzed the data, discussed the main structure and wrote the manuscript. All authors contributed to revisions and commented on previous versions of the manuscript.

### Conflict of interest statement

The authors declare that the research was conducted in the absence of any commercial or financial relationships that could be construed as a potential conflict of interest.
