# Supplementary material for: Combined Field Inoculations of Pseudomonas Bacteria, Arbuscular Mycorrhizal Fungi, and Entomopathogenic Nematodes and their Effects on Wheat Performance
Source: Front Plant Sci. 2017 Oct 31;8:1809. doi: 10.3389/fpls.2017.01809 (PMC5671467; doi:10.3389/fpls.2017.01809)
Supplement: Supplementary file 1 [file Presentation1.PDF]

Supplementary Table and Figures

Combined field inoculations of *Pseudomonas* bacteria, arbuscular mycorrhizal fungi and entomopathogenic nematodes and their effects on wheat performance

Nicola Imperiali†, Xavier Chiriboga†, Klaus Schlaeppi, Marie Fesselet, Daniela Villacrés, Geoffrey Jaffuel, S. Franz Bender, Francesca Dennert, Ruben Blanco-Pérez, Marcel G.A. van der Heijden, Monika Mauerhofer, Fabio Mascher, Ted C.J. Turlings, Christoph Keel, and Raquel Campos-Herrera*

†These authors have contributed equally to this work.

***Correspondence:**

Raquel Campos-Herrera, rcherrera@ualg.pt

Contents:

Table S1. Species and sources of nematodes, fungi and bacterium for the entomopathogenic nematode soil food web analysis by real time qPCR.

Figure S1. Experimental designs of the three field experiments performed in 2014 and 2015 at the agricultural research station Agroscope near Prangins, Switzerland.

FIGURE S2. Percentage of *P. protegens* CHA0 (B1) and *P. chlororaphis* PCL1391 (B2) isolated from wheat roots previously inoculated with the MIX treatment in the PERFORMANCE-2 field experiment.

Figure S3. Natural occurrence of free living nematodes (A, C and E) and nematophagous fungi (B, D, and F) in the three field experiments following application of entomopathogenic nematode.

Figure S4. Seedling survival after frit fly attack in the small AMF plots of the COMBINATION experiment.

Figure S5. Additional plant traits measured in the PERFORMANCE-2 experiment.

TABLE S1 | Species and sources of nematodes, fungi and bacterium for the entomopathogenic nematode soil food web analysis by real time qPCR.

Type of organism/ species	Population/strain	Material used / Unit of measurements	GenBank accession number[a]
Entomopathogenic nematodes			
Heterorhabditis bacteriophora	Commercial	Infective juvenile (IJs) / no. IJs	KJ938576
Heterorhabditis megidis	Commercial	Infective juvenile (IJs) / no. IJs	KJ938577
Steinernema affine	CH	Infective juvenile (IJs) / no. IJs	KJ938567
Steinernema carpocapsae	DOK-83	Infective juvenile (IJs) / no. IJs	KJ818295
Steinernema feltiae	RS-5	Infective juvenile (IJs) / no. IJs	KJ938569
Steinernema kraussei	OS	Infective juvenile (IJs) / no. IJs	KJ696686
Steinernema poinari	1160	ITS rDNA sequence + pUC57 / pg DNA	KF241754
Free-living and competitor nematodes			
Acrobeloides-group	RT1-R15C	18S rDNA sequence + pUC57 / pg of DNA	JQ237849
Oscheius tipulae	MG68 P29	Nematodes/ ng DNA	KJ938579
Oscheius onirici	MG67 P20	Nematodes/ ng DNA	KJ938578
Oscheius sp. 3	JU75	18S rDNA sequence + pUC57 / pg of DNA	AJ297890
Nematophagous fungi			
Catenaria sp.	1D	ITS rDNA sequence + pUC57 / pg of DNA	JN585805
Arthrobotrys dactyloides	H55	Pure culture / pg of DNA	KJ938574
Arthrobotrys musiformis	11	Pure culture / pg of DNA	KJ938572
Arthrobotrys oligospora	8	Pure culture / pg of DNA	KJ938573
Hirsutella rhossiliensis	2931	Pure culture / pg of DNA	n.a.
Purpureocillium lilacinus	9357	Pure culture / pg of DNA	KJ938575B
Ectophoretic bacteria			
Paenibacillus nematophilus	NEM2	16S rDNA sequence of 490 bp + pUC57 / copy numbers	AF480936

[a] n.a., not available.

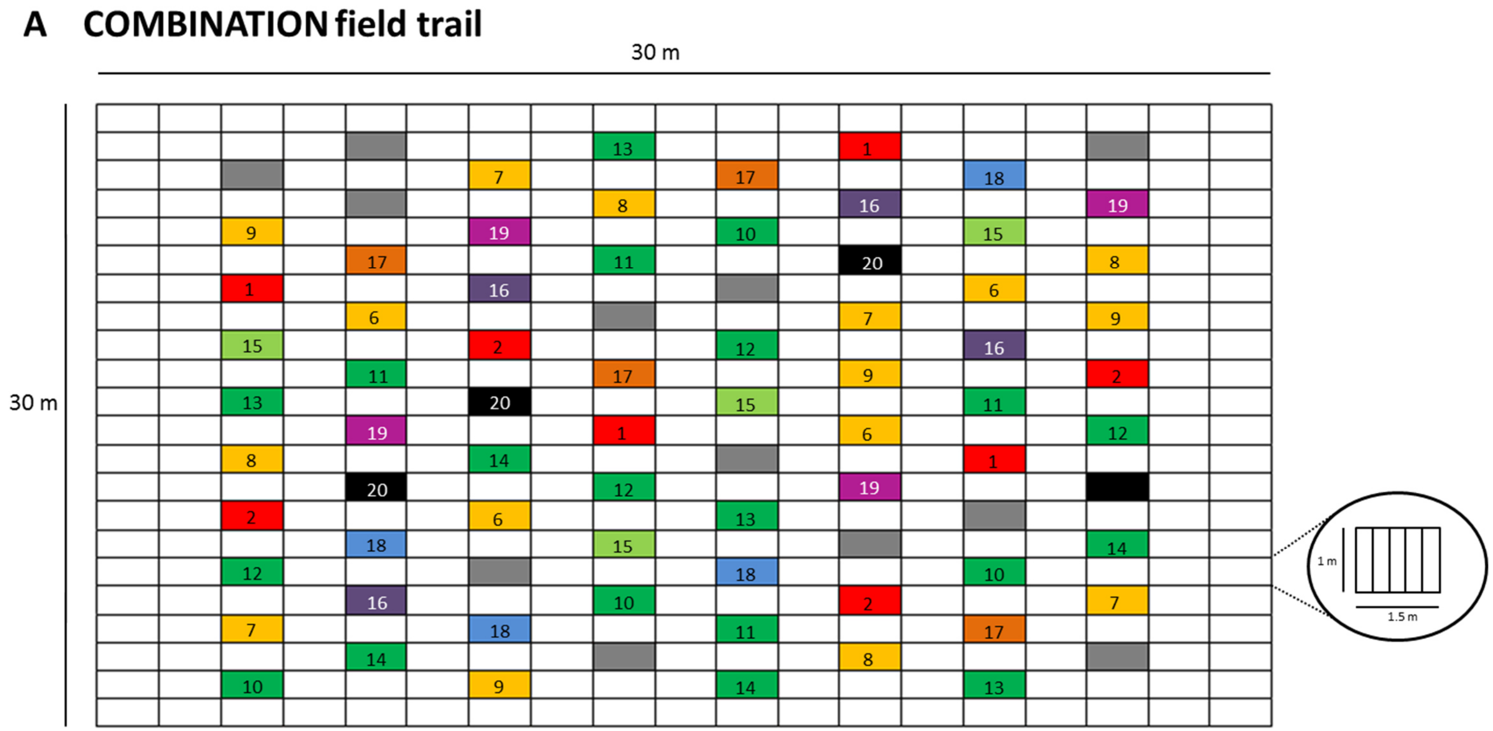

Bacteria	Nematodes	AMF	Combinations	Controls
1) *P. protegens* CHA0 (B1)	6) *H. megidis* Andermatt (N1)	10) *R. irregulare* INOQ Top (F1-H)	16) B1+ N2	20) Non inoculated plots
2) *P. chlororaphis* PCL1391 (B2)	7) *H. bacteriophora* Andermatt (N2)	11) *R. irregulare* INOQ Top (F1-L)	17) B1+ F1-H	Buffer
	8) *S. carpocapsae* D83 (N3)	12) *R. irregulare* SAF22 (F2)	18) N2+ F1-H	3,4,5) Not considered treatments
	9) *S. feltiae* RS5 (N4)	13) *F. mosseae* SAF11 (F3)	19) B1+ N2+ F1-H	
		14) *C. claroideum* SAF12 (F4)		
		15) AMF control		

B PERFORMANCE-1 field trail

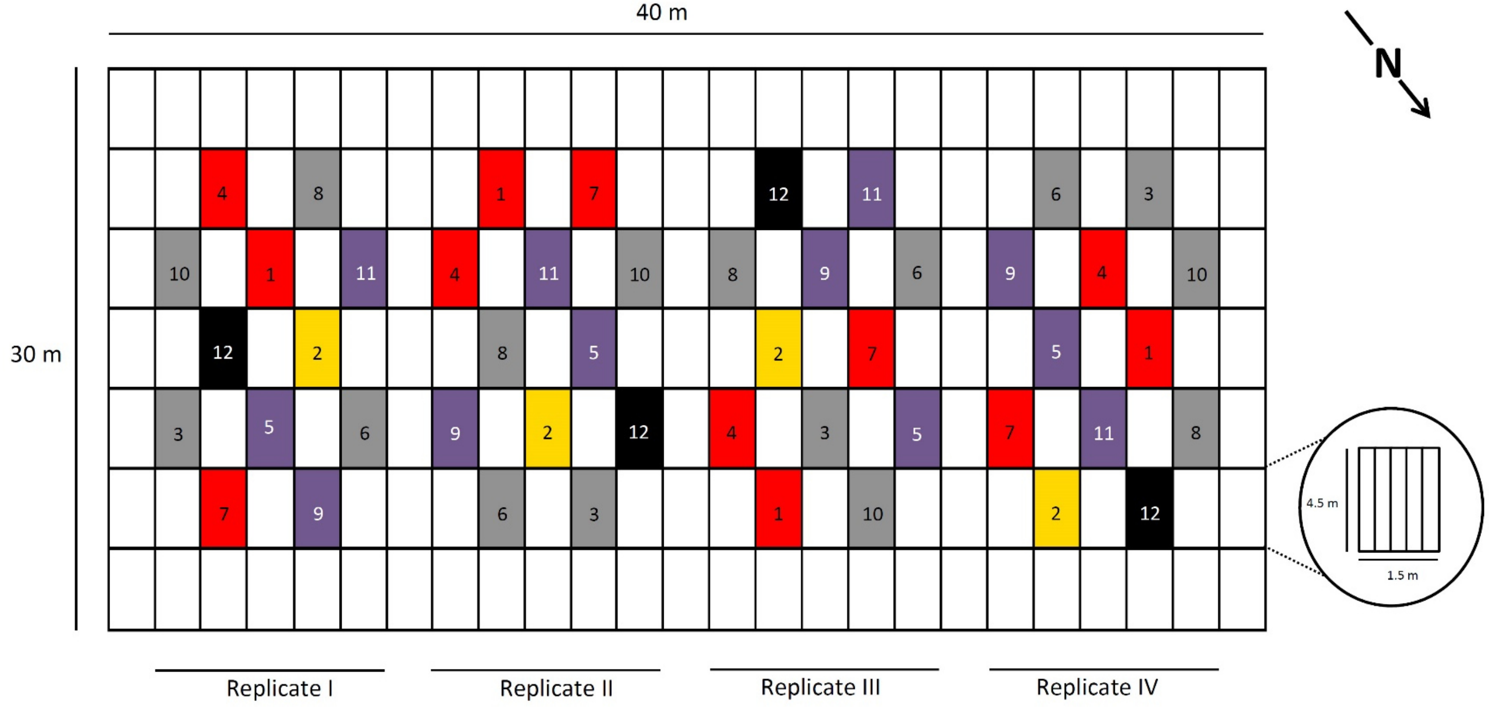

Bacteria	Nematodes	Combinations	Controls
1) *P. protegens* CHA0 (B1)	2) *H. bacteriophora* Andermatt (N2)	5) B1 + N2	12) Non inoculated plots
4) *P. chlororaphis* PCL1391 (B2)		9) B2 + N2	Buffer
7) *P. protegens* CHA0 + *P. chlororaphis* PCL1391 (BM)		11) BM + N2	3,6,8,10) Not considered treatments

C PERFORMANCE-2 field trail

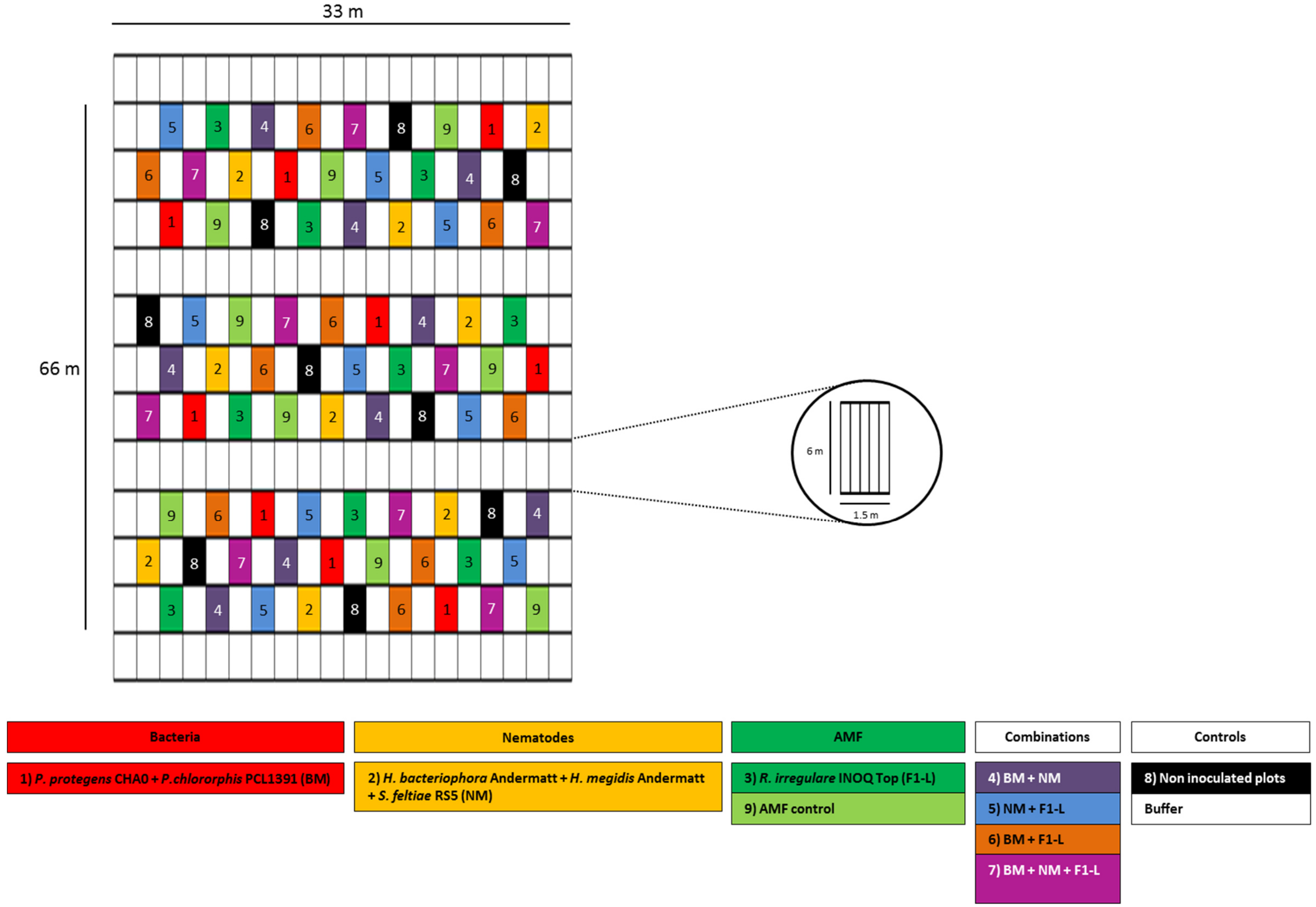

FIGURE S1 | Experimental designs of the three field experiments performed in 2014 and 2015 at the agricultural research station Agroscope near Prangins, Switzerland. Experiments were carried out using the spring wheat variety Rubli and individual or combined inoculations with rifampicin-resistant pseudomonads, entomopathogenic nematodes and arbuscular mycorrhizal fungi (AMF). Plots treated with different inoculants are indicated with different colours. Each plot consisted of five plant rows (row spacing of 50 cm). Inoculants were applied to the seed furrow immediately following sowing of wheat seeds. Non-coloured portions indicate buffer zones in which the triticale variety Trado was cultivated. Negative controls were untreated plots and, in addition for AMF, plots treated with a "mock inoculum" consisting of *Plantago* roots and substrate free of AMF propagules (AMF control). In each experiment, all replicates were disposed in Randomized Complete Block design. The COMBINATION field trial **(A)** conducted in spring 2014 included twenty treatments with four replicates per treatment. *Pseudomonas protegens* CHA0-Rif (B1) and *Pseudomonas chlororaphis* PCL1391-Rif (B2) were inoculated into the seed furrows of the 1.5 m^2 plots, individually or in combination with the entomopathogenic nematodes *Heterorhabditis megidis* Andermatt (N1), *Heterorhabditis bacteriophora* Andermatt (N2), *Steinernema carpocapsae* D83 (N3), *Steinernema feltiae* RS5 (N4) and the AMF strains *Rhizoglomus irregularis* INOQ Top*,* inoculated at high (i.e. 250 ml/row; (F1-H) and low concentration (i.e., 50 ml/row; (F1-L), *Rhizoglomus irregularis* SAF22 (F2), *Funneliformis mossae* SAF11 (F3) and *Glomus claroideum* SAF12 (F4). Grey-colored plots indicate three treatments (3, 4 and 5) with bacterial strains that were not considered for the present study. The PERFORMANCE-1 field experiment **(B)** was performed in spring 2014 and included four replicates of twelve treatments. *P. protegens* CHA0-Rif (B1) and *P. chlororaphis* PCL1391-Rif (B2) were inoculated into seed furrows of 6.75 m^2 plots, either individually, mixed (BM) or in combination with the entomopathogenic nematode *H. bacteriophora* Andermatt (N2). Grey-colored plots indicate four treatments (3, 6, 8 and 10) with application of chitosan that were not considered for the present study. The PERFORMANCE-2 field experiment **(C)** was conducted in spring 2015 and included nine replicates of nine treatments. For the different treatments, a mixture of the bacteria *P. protegens* CHA0-Rif and *P. chlororaphis* PCL1391-Rif (BM), a mixture of the entomopathogenic nematodes *H. megidis* Andermatt, *H. bacteriophora* Andermatt, and *S. feltiae* RS5 (NM), the AMF *R. irregularis* INOQ Top at 50 ml/row (F1-L) or combinations of the bacteria, nematodes, and AMF were inoculated into seed furrows of 9 m^2 plots.

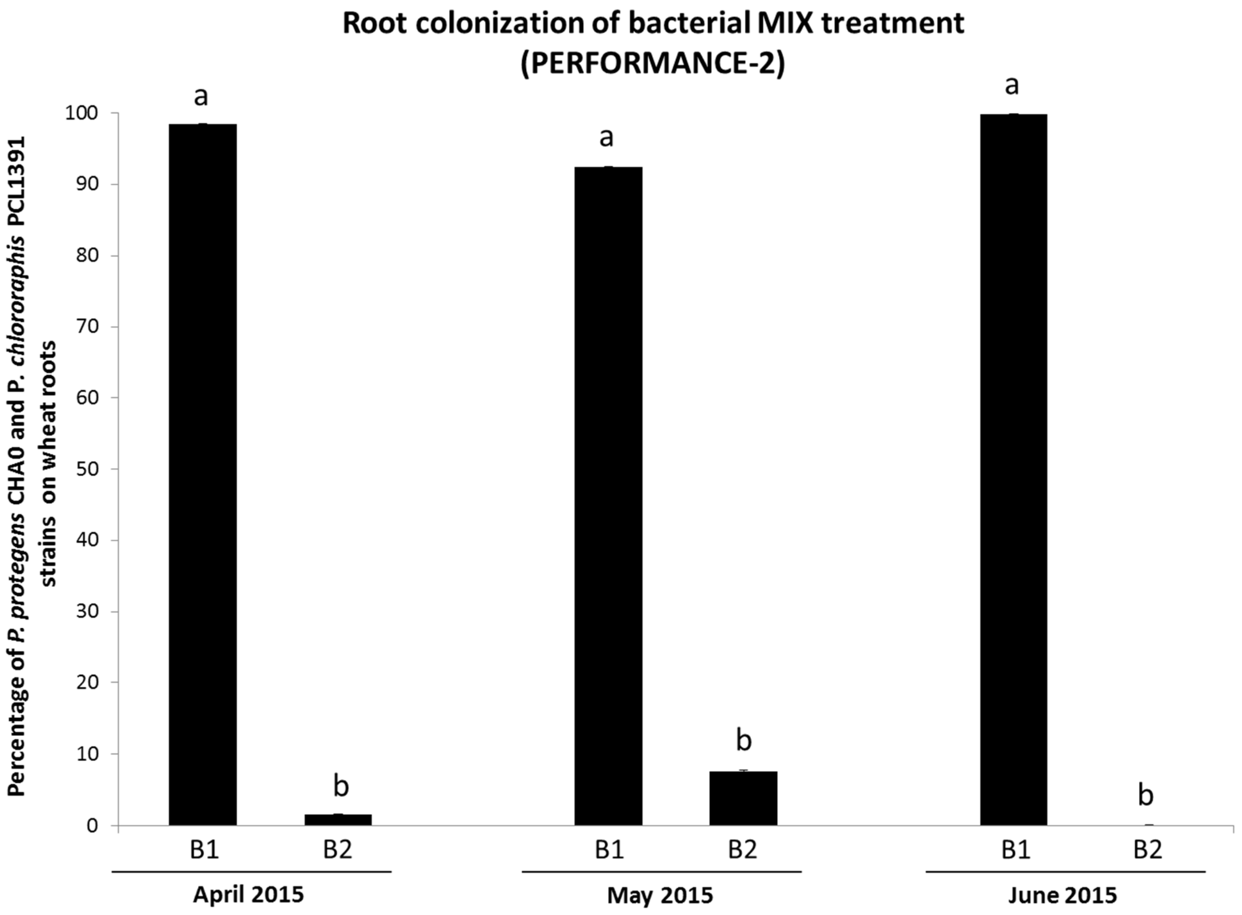

FIGURE S2 | Percentage of *P. protegens* CHA0 (B1) and *P. chlororaphis* PCL1391 (B2) isolated from wheat roots previously inoculated with the MIX treatment in the PERFORMANCE-2 field experiment. Inoculants were monitored by selective plating on KMB supplemented with rifampicin (100 µg/ml) and cycloheximide (100 µg/ml) at three different time points following seed furrow inoculation. Bar graphs show means of percentages of CFU of B1 and B2 observed on root washes from plots treated with bacteria (± SEM).

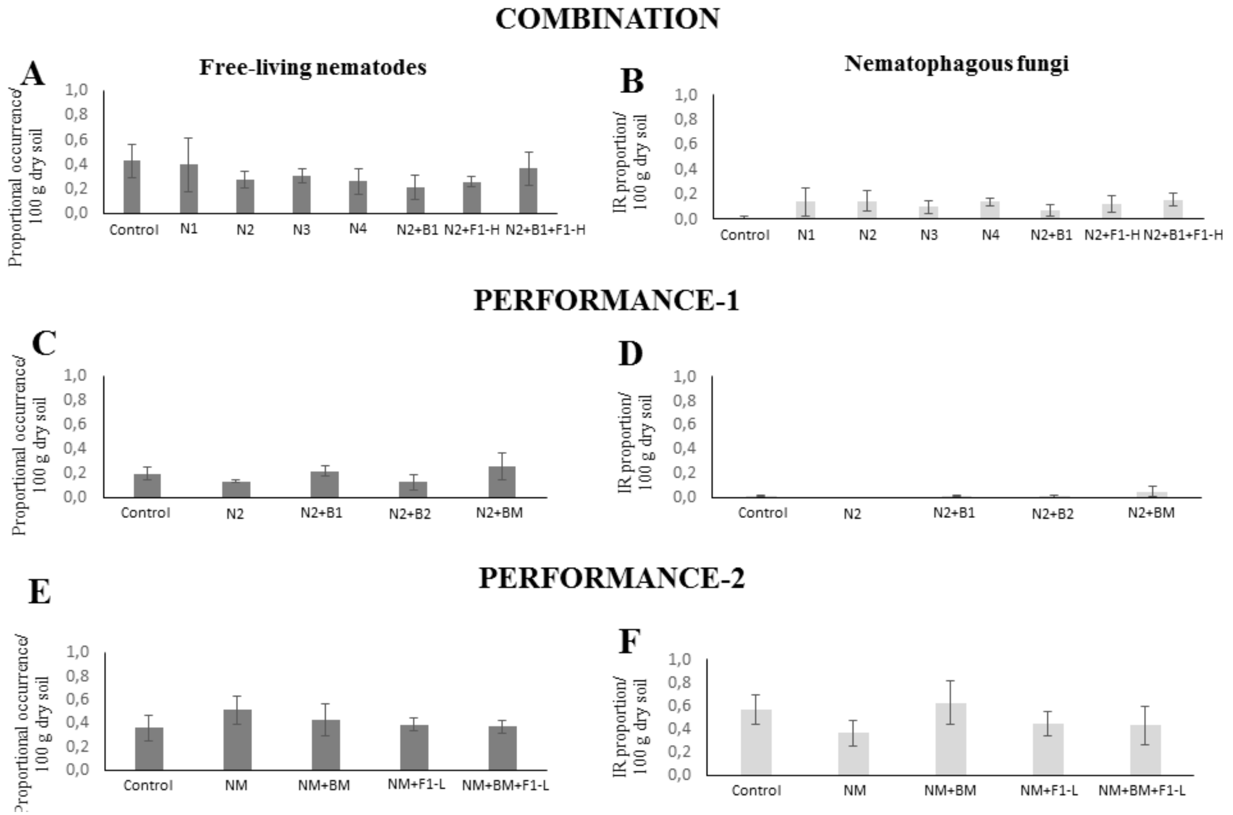

FIGURE S3 | Natural occurrence of free living nematodes (A, C and E) and nematophagous fungi (B, D, and F) in the three field experiments following application of entomopathogenic nematodes. Treatments: No inoculants (control), *H. megidis* Andermatt (N1), *H. bacteriophora* Andermatt (N2), *S. carpocapsae* D83 (N3), *S. feltiae* RS5 (N4), *P. protegens* CHA0 (B1), *P. chloraphis* PCL1391 (B2), AMF *R. irregularis* strain INOQ Top (high dosage, F1-H), AMF *R. irregularis* strain INOQ TOP (low dosage, F1-L), *P. protegens* CHA0 + *P. chloraphis* PCL1391 (BM), *H. megidis* Andermatt + *H. bacteriophora* Andermatt + *S. feltiae* RS5 (NM). The parasitism of nematodes by nematophagous fungi was determined by dividing the DNA quantity of each species by the total amount of DNA and expressed as “infection rate” (Campos–Herrera et al., 2012; Duncan et al., 2013). To estimate the total free-living nematodes and nematophagous fungi, we standardized the units of measurement among species to be on a scale of 0 to 1, by dividing all data within a species by the highest measurement for that species (de Rooij van der Goes et al., 1995). Both free-living nematodes and nematophagous fungi were expressed per 100 g of soil ± SEM. For statistical analysis, see details in Table 4.

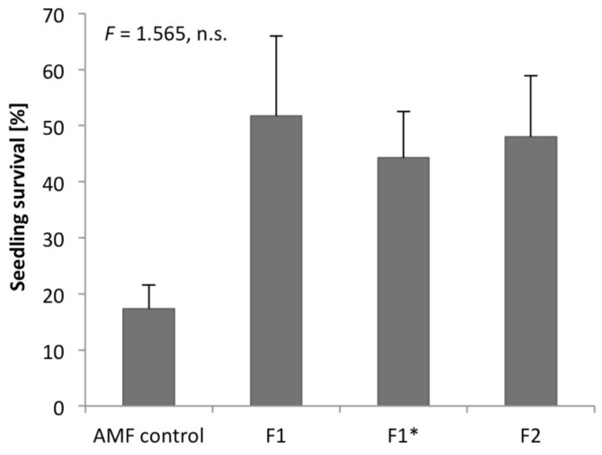

FIGURE S4 | Seedling survival after frit fly attack in the small AMF plots of the COMBINATION experiment. Treatments: AMF-free carrier (AMF control), AMF *Rhizoglomus irregularis* strain INOQ Top (high dosage, F1-H), AMF *Rhizoglomus irregularis* strain INOQ TOP (low dosage, F1-L) and AMF *Rhizoglomus irregularis* strain SAF22 (F2). Values presenting the mean (± SEM) plant density in the plots in percentages were analysed with one-way ANOVA (significance $P < 0.05$, n.s. not significant).

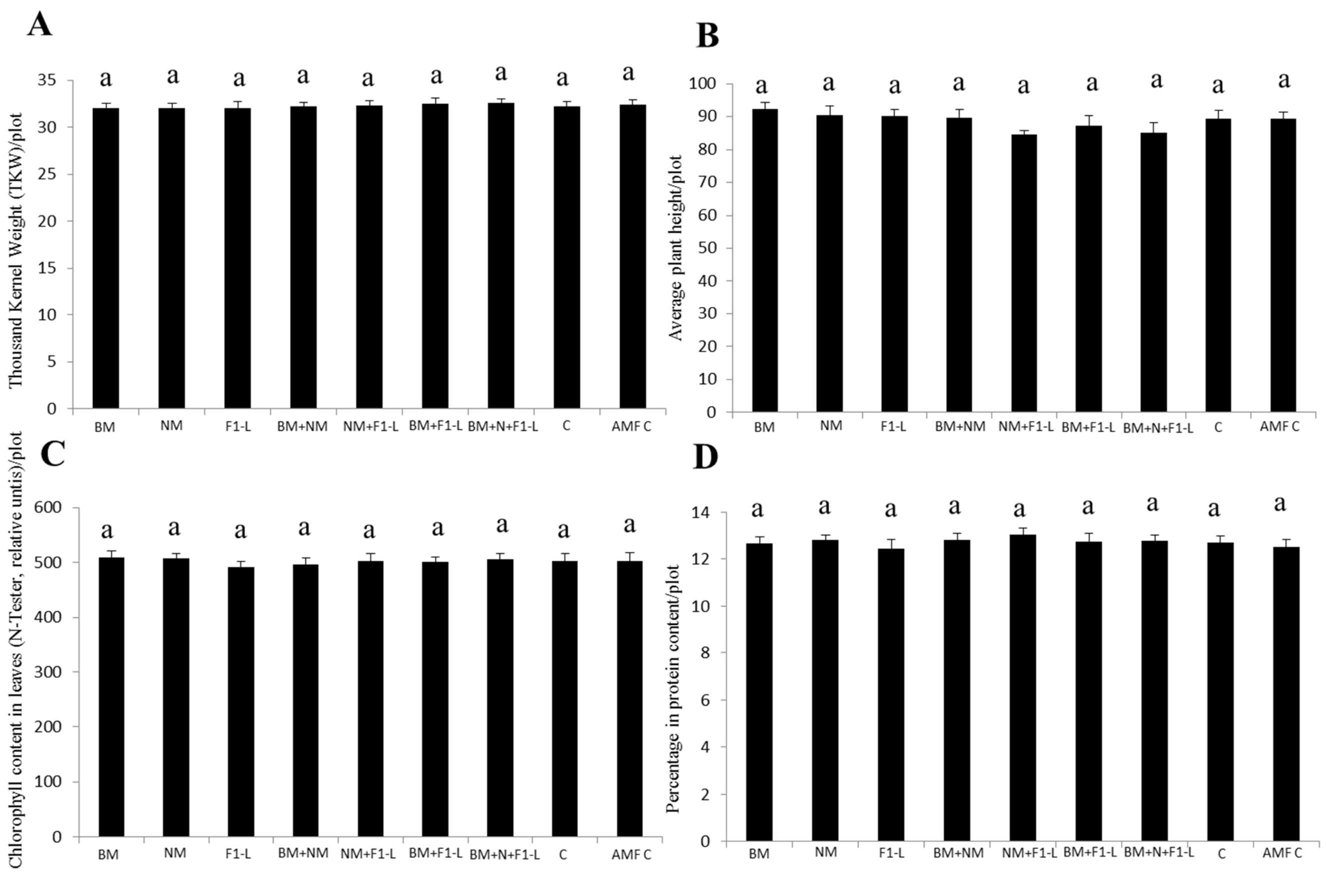

Supplementary data 4.

FIGURE S5 | Additional plant traits measured in the PERFORMANCE-2 experiment. Thousand kernel weight (TKW, expressed in g^{-1} per plot) values were determined by measuring the weight of wheat seeds in each plot (**A**). Plant height was measured from soil surface to the top of wheat plants, using a metre. Data are expressed as average plant height per plot (**B**). The chlorophyll content in wheat leaves was measured using a N-tester. Such data are strongly correlated with the state of nitrogen nutrition if the plant. Data are given in relative units per plot (**C**). Protein content in wheat plants were determined by infrared spectroscopy, and results are expressed in percentages per plot (**D**).
